# Supplementary material for: Geostatistical analysis and mapping of malaria risk in children of Mozambique
Source: PLoS One. 2020 Nov 9;15(11):e0241680. doi: 10.1371/journal.pone.0241680 (PMC7652261; doi:10.1371/journal.pone.0241680)
Supplement: S1 File — (PDF) [file pone.0241680.s001.pdf]

S1 File

Geostatistical Analysis and Mapping of Malaria Risk in Children of Mozambique

Bedilu Alamirie Ejigu,

Weighted Multilevel Modeling

Multilevel modeling has become a common way of model fitting procedure in large scale assessment surveys with multistage sampling like MMIS and unequal probability of selection [1, 2]. Failing to account for this aspect of the design can lead to biased parameter estimates [3–5]. To address this problem, analysts have recommended incorporating design weights in the likelihood function [2, 4–6]. Pfeffermann *et al* [6] developed a method for applying sampling weights to multilevel samples by defining a pseudo-likelihood using the following procedure.

1. Partition the weights by various levels of the model;
2. Normalize the weights at each level; and
3. Apply normalized weights to each level of the multilevel likelihood to create a pseudo likelihood.

In order to identify different risk factors, by taking in to account survey design weights, the following non-spatial multilevel mixed model is fitted to the data.

$$log(\frac{p_{ij}}{1-p_{ij}}) = \beta_o + x'_{ij}\beta + b_j, \tag{1}$$

In this model equation β_0 is an intercept, β is an unknown slope parameter for individual-level predictors, and b_j are mutually independent Gaussian random effects used to capture within-cluster correlation. In standard multilevel models, b_j is usually assumed to be a normally distributed random intercept with mean 0 and variance σ_b^2. Random intercepts models, like (Eqn 1) enable researchers to accommodate correlations within higher-level units resulting from clustered study designs. The weighted multilevel mixed model analyses which takes into account survey design weights was done using Stata 16.1 [7].

S1 Table. Parameter estimates from the multilevel model (1) of malaria prevalence in children under five years of age in Mozambique.

Factors	AOR	95% CI
Age (in month)	1.012	(1.006,1.018)
Bednet (yes)	0.846	(0.511,1.402)
Anemia	2.590	(1.994,3.365)
**Wealth index(Poorest)**		
Poor	0.874	(0.644,0.1.185)
Middle	0.661	(0.459,0.949)
Rich	0.362	(0.186,0.708)
Richest	0.108	(0.038,0.309)
**Education (No education)**		
Primary	0.591	(0.591,1.066)
Secondary/higher	0.356	(0.356,1.234)
**Residence (urban)**		
Rural	1.065	(0.840,1.291)
**Province(Cabo Delgado)**		
Niassa	0.599	(0.330,1.087)
Nampula	0.725	(0.339,1.552)
Zamboza	0.588	(0.321,1.078)
Tete	0.203	(0.073,0.563)
Manica	0.706	(0.327,1.525)
Sofala	0.366	(0.159,0.840)
Inhambane	0.639	(0.295,1.385)
Gaza	0.201	(0.083,0.483)
Maputo Province	0.034	(0.006,0.721)
Maputo City	0.134	(0.025,0.721)
**ITN coverage**	0.999	(0.999,1.000)
**Malaria incidence**	5.103	(1.000,26.123)
Var(b_j)	0.844	(0.541,1.318)

AOR stands for adjusted odds ratio, and CI for confidence interval.

References

1. Rabe-Hesketh S, Skrondal A. Multilevel modelling of complex survey data. Royal Statistical Society (Series A). 2006;169(4):805–827.

2. Carle AC. Fitting multilevel models in complex survey data with design weights: recommendations. BMC Medical Research Methodology. 2009;9:49–62.

3. Asparouhov T. General Multi-Level Modeling with Sampling Weights. Communications in statistics -Theory and methods. 2006;35(3):93–103.

4. Grilli L, Pratesi M. Weighted estimation in multilevel ordinal and binary models in the presence of informative sampling designs. Survey Methodology. 2004;30:93–103.

5. Pfeffermann D. Modelling of complex survey data: why mode? Why is it a problem? How can we approach it? Survey Methodology. 2011;37:115–136.

6. Pfeffermann D, Skinner CJ, Holmes DJ, Goldstein H, Rasbash J. Weighting for unequal selection probabilities in multilevel models. Royal Stat Soc. 1998;60:23–40.

7. StataCorp. Stata Statistical Software: Release 14; 2015. Available from: `https://www.stata.com/`.
