# Supplementary material for: Geostatistical analysis and mapping of malaria risk in children of Mozambique
Source: PLoS One. 2020 Nov 9;15(11):e0241680. doi: 10.1371/journal.pone.0241680 (PMC7652261; doi:10.1371/journal.pone.0241680)
Supplement: Attachment — Submitted filename: PONE-D-20-18063_R1_reviewer - seen.pdf [file pone.0241680.s002.pdf]

PLOS ONE

Geostatistical Analysis and Mapping of Malaria Risk in Children of Mozambique

--Manuscript Draft--

**Manuscript Number:**	PONE-D-20-18063R1
**Article Type:**	Research Article
**Full Title:**	Geostatistical Analysis and Mapping of Malaria Risk in Children of Mozambique
**Short Title:**	Geostatistical Modeling of Malaria in Children of Mozambique
**Corresponding Author:**	Bedilu Alamirie Ejigu, Ph.D<br>Addis Ababa University College of Natural Sciences<br>Addis Ababa, ETHIOPIA
**Keywords:**	geostatistics; Malaria; Mozambique; Spatial autocorrelation
**Abstract:**	Malaria remains one of the most prevalent infectious diseases in the tropics and subtropics, and Mozambique is not an exception. To design geographically targeted and effective intervention mechanisms of malaria, an up-to-date map that shows the spatial distribution of malaria is needed. This study analyzed 2018 Mozambique Malaria Indicator Survey using geostatistical methods to: i) explore individual, household, and community-level determinants of malaria in under-five children, ii) prepare a malaria prevalence map in Mozambique, and iii) produce prediction prevalence maps and exceedence probability across the country. The results show the overall weighted prevalence of malaria was 38.9% (N=4347, with 95% CI: 36.9% - 40.8%). Across different provinces of Mozambique, the prevalence of malaria ranges from 1% in Maputo city to 57.3% in Cabo Delgado province. Malaria prevalence was found to be higher in rural areas, increased with child's age, and decreased with household wealth index and mother's level of education. Given the high prevalence of childhood malaria observed in Mozambique there is an urgent need for effective public health interventions in malaria hot spot areas. The household determinants of malaria infection that are identified in this study as well as the maps of parasitaemia risk could be used by malaria control program implementers to define priority intervention areas.
**Order of Authors:**	Bedilu Alamirie Ejigu, Ph.D
**Response to Reviewers:**	Review Comments to the Author<br><br>Reviewer #1: I would like to commend the authors for very good and detailed analysis. A wealth of information has been presented in this paper including a comparison between neighboring countries. All my comments are quite straight forward<br><br>Major comments<br><br>1. I have noticed that figure 4 is not adequately described especially in the discussion. It would be great to pinpoint the areas with probabilities above the threshold and explain why that is the case, i.e. link the map with actual locations. The authors should be bold enough in the discussion section to speculate why some areas have high observed probabilities, what are the characteristics of those areas etc. On the same note, it is not clear how the 20% threshold was chosen. Is it a government of Mozambique or WHO standard? This must be made clear.<br>-In the revised version of the manuscript, additional description was provided in the result and discussion section which considerably strength the paper.<br>-In order to guide model-based geostatistical predictions, thresholds of risks were used that resulted in 20% of the population being included in the hotspot based on the theoretical 80–20 assumption where 20% of the population constitutes 80% of the exposure and transmission events (Woolhouse et al 1997, Stresman et al 2017). This threshold selection is likely to be location specific and the hotspot sizes will vary based on the threshold selected: a high threshold would result in only those areas with the highest transmission being identified as a hotspot and a more granular map whereas a less stringent threshold would mean that hotspots would be more ubiquitous. As implemented by different authors (Stresman et al 2017, Chipeta et al 2019,Yankson et al 2019), it common approach to consider 20% as a threshold in the analysis of malaria data.

Powered by Editorial Manager® and ProduXion Manager® from Aries Systems Corporation

	2. Two major modelling methods are presented here. I thought that the non-spatial approach was done to select the variables to go into the geostatistical model. Table 3 gives the impression that the intention was to compare the 2 approaches. The model-fitting approach should be clearly spelt out in the methods section to avoid any confusion.<br>- As you noticed, non-spatial multilevel model were fitted to identify significant variables by taking into account the sampling weight. Since the focus in this study is to analyze 2018 MMIS using model based geostatistics, to avoid confusion I presented results only obtained using geostatistical model. As a result, descriptions of multilevel modeling from the methods section and parameter estimates from the result table were dropped.<br>3. Varying number of decimal points are presented throughout the paper – sometimes 2 and in most places 3 decimal places. In Table 2, we have 1 decimal place. This must be standardized<br>-This issue addressed throughout the revised manuscript.<br>4. Discussion section – Are there any limitations associated with this study? You make no mention of any possible limitations. Related to this point, you could also layout future areas of research to address any limitations.<br>- Additional paragraph mentioning limitations associated with this study included in the revised version.<br>Minor comments<br><br>1. Line 43 – the word "doubling" is very specific and sounds as if it's based on empirical evidence. We are simply not sure about the effects of covid on malaria. Instead of doubling, I suggest use a less specific term such as "…increase in the number of malaria deaths…"<br>- Corrected.<br>2. Line 44 – This line "…affected countries like Mozambique could identify malaria hot-spot areas and move…" the word "could " in this sentence seems misplaced and as such, sentence is not very clear<br>-Corrected.<br><br>3. Section 2.1 – it is okay that you have referenced the reader to the survey final report for details on the methods. However, I feel at least a paragraph summarizing the methods should be included before referring the reader to the report for the rest of the details. Since in this paper you have fitted multilevel models that take into account the clustering, a short description would be nice. You can limit this description to the part of the sampling methodology that introduces the clusters.<br>- Additional description about survey methodology included.<br>4. Line 137, use lower case for "Model"<br>- Corrected.<br>5. Table 1 – Is it Zamboza or Zambezia province? Please check<br>-Corrected as Zambezia.<br>6. Table 1 – Until this table, there is no mention of MIS 2011 in the previous section. Shouldn't it mention somewhere in the methods that there will be comparison between the 2 time points and also comparison between neighbouring countries?<br>- A short description included in the methods Section.<br>7. Table 1: Mother education label – remove the word "label"<br>-Corrected.<br>8. Table 1: HH Mosequto bednet – wrong spelling for mosquito<br>-Corrected.<br><br>9. Table 1: Child age in year – make is concise – something like "child age (years)"<br>-Corrected.<br><br>10. Line 196 - … geostatistical modeling part an exponential function was assumed - Not clear what the exponential function was assumed for. Is for the correlation function. Earlier, you mentioned that a matern function was assumed. Could you please clarify?<br>-Yes, it is for the correlation function, and corrected accordingly. Exponential correlation function is a special case of Matern function when k=1/2.<br><br>11. Line 197 - The assumption provide to be correct as the correlation – I guess it should be "… the assumption proved to be correct …"<br>- Corrected.

Powered by Editorial Manager® and ProduXion Manager® from Aries Systems Corporation

	12. Line 204 – The sentence “The likelihood of being parasitaemia for children living with household wealth index” does not sound very well. I suspect you are missing a word.<br>-Yes, corrected.<br><br>14. Table 3 – swap the columns so that you start with non-spatial model (3) and then spatial model (4)<br>- As stated earlier, to avoid confusion, results from multilevel model (3) dropped.<br>15. From line 244 in the discussion – the paragraph mentions 2 provinces with high prevalence and you mention that prevalence varies across socio-demographic groups. Can you speculate further as to why these 2 provinces have the highest prevalence? What is their socio-demographic profile in comparison with the others? This will make the discussion richer.<br>- Thanks. The following statement included in the revised version.<br>“…..This may be due to the fact that, the most populous provinces Zambezia and Nampula have the worst education and health outcomes; and in general northern provinces have worse infrastructure; higher levels of environmental degradation and less economic activity than the south \cite{SIDA, 2019}. Further, a secondary school access is unevenly distributed among the provinces and lowers in Cabo Delgado and Niassa province (Fox et al, 2019). “
**Additional Information:**	
**Question**	**Response**
**Financial Disclosure**<br><br>Enter a financial disclosure statement that describes the sources of funding for the work included in this submission. Review the submission guidelines for detailed requirements. View published research articles from *PLOS ONE* for specific examples.<br><br>This statement is required for submission and **will appear in the published article** if the submission is accepted. Please make sure it is accurate.	The author received no specific funding for this work.

**Unfunded studies**<br>Enter: *The author(s) received no specific funding for this work.*<br><br>**Funded studies**<br>Enter a statement with the following details:<br>• Initials of the authors who received each award<br>• Grant numbers awarded to each author<br>• The full name of each funder<br>• URL of each funder website<br>• Did the sponsors or funders play any role in the study design, data collection and analysis, decision to publish, or preparation of the manuscript?<br>• **NO** - Include this sentence at the end of your statement: *The funders had no role in study design, data collection and analysis, decision to publish, or preparation of the manuscript.*<br>• **YES** - Specify the role(s) played.<br><br>* typeset	
**Competing Interests**<br><br>Use the instructions below to enter a competing interest statement for this submission. On behalf of all authors, disclose any competing interests that could be perceived to bias this work—acknowledging all financial support and any other relevant financial or non-financial competing interests.<br><br>This statement **will appear in the published article** if the submission is accepted. Please make sure it is accurate. View published research articles from *PLOS ONE* for specific examples.	The authors have declared that no competing interests exist.

**NO authors have competing interests**<br><br>Enter: *The authors have declared that no competing interests exist.*<br><br>**Authors with competing interests**<br><br>Enter competing interest details beginning with this statement:<br><br>*I have read the journal's policy and the authors of this manuscript have the following competing interests: [insert competing interests here]*<br><br>* typeset	
**Ethics Statement**<br><br>Enter an ethics statement for this submission. This statement is required if the study involved:<br><br>• Human participants<br>• Human specimens or tissue<br>• Vertebrate animals or cephalopods<br>• Vertebrate embryos or tissues<br>• Field research<br><br>Write "N/A" if the submission does not require an ethics statement.<br><br>General guidance is provided below. Consult the submission guidelines for detailed instructions. **Make sure that all information entered here is included in the Methods section of the manuscript.**	N/A

**Format for specific study types**<br><br>**Human Subject Research (involving human participants and/or tissue)**<br>• Give the name of the institutional review board or ethics committee that approved the study<br>• Include the approval number and/or a statement indicating approval of this research<br>• Indicate the form of consent obtained (written/oral) or the reason that consent was not obtained (e.g. the data were analyzed anonymously)<br><br>**Animal Research (involving vertebrate animals, embryos or tissues)**<br>• Provide the name of the Institutional Animal Care and Use Committee (IACUC) or other relevant ethics board that reviewed the study protocol, and indicate whether they approved this research or granted a formal waiver of ethical approval<br>• Include an approval number if one was obtained<br>• If the study involved *non-human primates*, add *additional details* about animal welfare and steps taken to ameliorate suffering<br>• If anesthesia, euthanasia, or any kind of animal sacrifice is part of the study, include briefly which substances and/or methods were applied<br><br>**Field Research**<br><br>Include the following details if this study involves the collection of plant, animal, or other materials from a natural setting:<br>• Field permit number<br>• Name of the institution or relevant body that granted permission	
**Data Availability**<br><br>Authors are required to make all data underlying the findings described fully available, without restriction, and from the time of publication. PLOS allows rare exceptions to address legal and ethical concerns. See the PLOS Data Policy and FAQ for detailed information.	No - some restrictions will apply

Powered by Editorial Manager® and ProduXion Manager® from Aries Systems Corporation

A Data Availability Statement describing where the data can be found is required at submission. Your answers to this question constitute the Data Availability Statement and **will be published in the article**, if accepted.<br><br>**Important:** Stating 'data available on request from the author' is not sufficient. If your data are only available upon request, select 'No' for the first question and explain your exceptional situation in the text box.<br><br>Do the authors confirm that all data underlying the findings described in their manuscript are fully available without restriction?	
**Describe where the data may be found in full sentences. If you are copying our sample text, replace any instances of XXX with the appropriate details.**<br><br>• If the data are **held or will be held in a public repository**, include URLs, accession numbers or DOIs. If this information will only be available after acceptance, indicate this by ticking the box below. For example: *All XXX files are available from the XXX database (accession number(s) XXX, XXX.)*.<br>• If the data are all contained **within the manuscript and/or Supporting Information files**, enter the following: *All relevant data are within the manuscript and its Supporting Information files.*<br>• If neither of these applies but you are able to provide **details of access elsewhere**, with or without limitations, please do so. For example:<br><br>*Data cannot be shared publicly because of [XXX]. Data are available from the XXX Institutional Data Access / Ethics Committee (contact via XXX) for researchers who meet the criteria for access to confidential data.*<br><br>*The data underlying the results presented in the study are available from (include the name of the third party*	The data we used which is the '2018 Mozambique Malaria Indicator Survey' were obtained from the DHS program (www.dhsprogram.com), but the 'Dataset Terms of Use' do not permit us to distribute this data as per data access instructions (http://dhsprogram.com/data/Access-Instructions.cfm). To get access for the dataset you must first be a registered user of the website (www.dhsprogram.com), and download the 2018 Mozambique Malaria Indicator Survey. Interested scholars will have access to the relevant data used in this study in the same manner as it was accessed by the authors of this study from the aforementioned website.

Powered by Editorial Manager® and ProduXion Manager® from Aries Systems Corporation

and contact information or URL).<br>• This text is appropriate if the data are owned by a third party and authors do not have permission to share the data.<br><br>* typeset	
Additional data availability information:	

Manuscript Click here to access/download;Manuscript;Manuscript.pdf

Geostatistical Analysis and Mapping of Malaria Risk in Children of Mozambique

Bedilu Alamirie Ejigu[1*],

1 Department of Statistics, College of Natural and Computational Sciences, Addis Ababa University, Addis Ababa, Ethiopia.

* Corresponding author
E-mail: bedilu.alamirie@aau.edu.et (B.A Ejigu)

abstract

Malaria remains one of the most prevalent infectious diseases in the tropics and subtropics, and Mozambique is not an exception. To design geographically targeted and effective intervention mechanisms of malaria, an up-to-date map that shows the spatial distribution of malaria is needed. This study analyzed 2018 Mozambique Malaria Indicator Survey using geostatistical methods to: i) explore individual, household, and community-level determinants of malaria in under-five children, ii) prepare a malaria prevalence map in Mozambique, and iii) produce prediction prevalence maps and exceedence probability across the country. The results show the overall weighted prevalence of malaria was 38.9% (N=4347, with 95% CI: 36.9% - 40.8%). Across different provinces of Mozambique, the prevalence of malaria ranges from 1% in Maputo city to 57.3% in Cabo Delgado province. Malaria prevalence was found to be higher in rural areas, increased with child's age, and decreased with household wealth index and mother's level of education. Given the high prevalence of childhood malaria observed in Mozambique there is an urgent need for effective public health interventions in malaria hot spot areas. The household determinants of malaria infection that are identified in this study as well as the maps of parasitaemia risk could be used by malaria control program implementers to define priority intervention areas.

1 Background

Malaria is an infectious disease caused by a parasite that is transmitted from one subject to another by blood-sucking female anopheles mosquitoes. It is a major public health problem, especially in Africa and Asia. Many countries made an incredible progress in the fight against malaria, and as a result malaria deaths have fallen by more than 50% globally between 2000 and 2015. Seventeen countries eliminated malaria, and six were certified by WHO as malaria-free ([1]). Based on WHO recent report, on a global scale, progress has levelled off but no gains were achieved in reducing malaria case incidence over the last five years ([2]). The WHO strategic advisory group predicts that there will be 11 million cases of malaria in Africa by 2050 ([2]). The current COVID-19 pandemic places extra burden on health systems worldwide, and especially in sub-Saharan Africa which accounts for more than 90% of global malaria cases and deaths. Recently, WHO recommends, countries to move quickly to save lives from malaria in sub-Saharan Africa ([1]).

In Africa, more than two-thirds of all malaria deaths occur in children under-five years old ([3]). Because of a continual fight against malaria by intervention programs,

malaria infection prevalence and clinical incidence decreased by 50% and 40%, between 2000 and 2015, respectively ([4]). In 2017, an estimated 219 million cases and 435 thousand deaths of malaria occurred worldwide, of which 200 million (92%) malaria cases were in the WHO African Region ([3]). Fifteen sub-Saharan Africa countries and India carried 80% of the global malaria burden and Mozambique accounts for 5%. Children under 5 years of age are the most vulnerable group affected by malaria and they accounted for 61% of all malaria deaths worldwide ([3]).

In Mozambique, malaria is a common disease with seasonal fluctuation in all parts of the country with seasonal peak ranging from December to April ([5]). The prevalence varies across different ecological zones and transmission occurs year-round with relatively higher prevalence in the northern part of the country. Various factors influence the dynamics of malaria transmission and infection ranging from natural (i.e. rainfall, temperature) to social factors. Previously conducted national surveys showed that the prevalence of malaria in under five children was 39% and 40% in the years 2011 and 2015, respectively. Malaria accounts for 29% of all deaths and 42% of deaths in under-five children in the country ([6]).

Even though, Mozambique's entire population is at risk of malaria due to different environmental and ecological factors ([7]), pregnant women and under-five children are at higher risk of severe illness due to their low immunity ([8–10]). Thus, efficient interventions and preventive measures could be improved by advancing our understanding of the spatial patterns of malaria prevalence distribution and the underlying factors. Long-lasting insecticidal nets (LLINs) is one of the main interventions mechanisms for preventing malaria infection, and the 2017-2022 Mozambique strategic plan aims to achieve one net for every two people LLINs coverage across the country ([6]). Currently, disruptions to insecticide-treated net campaigns due to COVID-19 pandemic and in access to antimalarial medicines could lead to increase in the number of malaria deaths and co-morbidities. This requires malaria affected countries like Mozambique should identify malaria hot-spot areas and move quickly to save lives from malaria. The spatial distribution risk map of malaria is an important tool for effective planning, malaria control intervention, resource mobilization, monitoring and evaluation process. As a result, to advance intervention mechanisms, spatial distribution maps of malaria prevalence across the study area have been produced using geostatistical modeling approaches ([11–21]) with the aim of identifying areas where greatest control effort should be focused. Previously generated maps depicted the geographical distribution of malaria risk in Mozambique either at province or continental-scale ([4, 7, 22–25]), not at country-level. However, those maps may not reflect the current malaria situation in the country, as they rely on historical and outdated survey data. Malaria risk maps based on historical data cannot reflect the current situation which is changing due to ongoing interventions.

To date, to our knowledge, the only map available for the spatial distribution of malaria prevalence in Mozambique based on recent data was produced for Chimoio ([7]) and Maputo province ([5, 22]) which do not represent the current situation since it does not take into account contemporary effects of interventions and socio-economic status. Further, recently produced reports related with malaria in Mozambique do not address the spatial distribution of malaria across survey clusters of the country by taking into account other factors ([6, 26]).

In this study, the 2018 Mozambique Malaria Indicator Survey (MMIS) data were analyzed using geostatistical modeling approaches to: i) identify determinant factors associated with malaria risk, ii) produce a prevalence map of malaria among children under the age of five across survey clusters and regions of Mozambique, and iii) produce prediction prevalence maps and exceedence probability across the country. Predicted malaria prevalence maps generated in this study would help policy makers to identify

high-risk areas and design targeted interventions.

2 Materials and Methods

2.1 Mozambique MIS Data

The data for this study were obtained from the 2018 Mozambique Malaria Indicator Survey (MMIS). The main aim of this survey was to obtain population-based estimates of malaria indicators by considering a nationally representative dataset which serves as input for strategic planning and evaluation of malaria control program([26]). Stratified two-stage sampling technique was used to select enumeration areas (EAs) and households. Sampling procedures of the survey have been mentioned in the survey final report ([26]). Permission to use the 2018 MMIS data was obtained from the DHS website (`www.dhsprogram.com`). Figure 1 (left panel) presents the map of survey cluster locations where raw dataset were collected, and prevalence is depicted (right panel).

The geostatistical modeling includes: individual-level variables:child age, gender, anaemia level, child slept under bed net; household-level variables including educational level of the mother, household wealth index, and availability of bed nets in the household; and community-level variables: place of residence, mean temperature, estimated malaria incidence in the cluster, ITN coverage, and region were used in the analysis. Cluster-level geospatial data used in this study (ITN-coverage, malaria incidence) were obtained from DHS Program Geospatial Covariate Datasets (`www.dhsprogram.com`), and the construction procedures of geospatial data were explained in [27]. Further, to see the change in the prevalence of malaria by different factors in the past seven years, the 2011 Mozambique demographic and health survey used [28]. In addition to this, to compare the overall risk of malaria with neighboring countries, nationwide malaria prevalence in Tanzania, Malawi, Zambia, and Zimbabwe considered.

2.2 Statistical Analysis

Malaria indicator survey data-sets are often complex in nature for two reasons: i) the use of stratified multistage cluster sampling to increase sampling and cost efficiency, and ii) unequal probabilities of selection from target-populations for sampled elements, often as a result of oversampling of key subgroups. Thus, our data analysis tools employed sampling weights for generating unbiased population estimates ([29]).

2.2.1 Weighted Confidence Interval for Proportions

Constructing a confidence interval for proportion p is one of the most basic analyses in statistical inference, and it is an important aspect of reporting statistical results. Let Y denote a binomial variate for sample size n, and let $\hat{p} = \frac{Y}{n}$ denote the sample proportion. Under asymptotic normality of the sample proportion and estimating the standard error, an approximate $100(1-\alpha)\%$ confidence interval for P is

$$\hat{p} \mp z_{1-\alpha/2}\sqrt{\hat{p}(1-\hat{p})/n}. \tag{1}$$

The large-sample Wald intervals (Eqn 1) are known to perform poorly ([30]), but the Wilson intervals ([31]) given by Eqn 2 have been shown to perform well in a variety of situations.

$$\frac{\hat{p} + z^2_{1-\alpha/2}/2n}{1 + z^2_{1-\alpha/2}/n} \mp \frac{z_{1-\alpha/2}\sqrt{\hat{p}(1-\hat{p}) + z^2_{1-\alpha/2}/4n}}{\sqrt{n}(1 + z^2_{1-\alpha/2}/n)} \tag{2}$$

Both Wald (Eqn 1) and Wilson (Eqn 2) intervals are appropriate for survey data with simple random sampling designs, but they are not designed to accommodate clustering or unequal weights of more complex sample surveys, like the data analyzed in this manuscript.

A common approach to construct confidence intervals for proportions from complex sample survey data is to modify the inputs of Eqn 2 to account for survey weights and the design effect. The survey-weighted, estimated proportion,$\hat{p}$, is used along with a consistent design-based estimate, $\hat{Var}(\hat{p})$, of its variance. For complex survey data. [32] propose a modified version by replacing $z_{1-\alpha/2}$ with $t_c(1-\alpha/2)$ in equation Eqn 2, and replacing n with the effective sample size, defined as $n_{eff} = n/\hat{Deff}(\hat{p})$ where

$$\hat{Deff}(\hat{p}) = \frac{\hat{Var}(\hat{p})}{\hat{p}(1-\hat{p})/n} = \frac{\sum_h (\frac{N_h}{N})^2 (1-\frac{n_h}{N}) \frac{\hat{p}(1-\hat{p})}{n_h}}{\hat{p}(1-\hat{p})/n} = \frac{n\sum_i^n w_i^2}{(\sum_i^n w_i)^2}$$

as the estimated design effect ([33]), w_i is the weight of the ith unit selected in the sample, w represents sampling weights that denote the inverse of the probability that the observation is included because of the sampling design.

2.2.2 Geostatistical Modeling

Non-spatial modelling approaches assume independence between study locations where the data collected inadvertently neglect potential spatial dependency between neighboring locations due to unobserved common exposures. To overcome such limitations, geostatistical models relate disease prevalence data with potential predictors and quantify spatial dependence via the covariance matrix of a Gaussian process facilitated by adding random effects at the observed locations ([39]). Such type of geostatistical models have already been applied to model malaria risk at different geographical scales in different Africa countries ([11–13, 15, 19, 20, 40]).

In model equation 3 below, malaria status Y_{ij} of child i at location j take a value of 1 if the child has malaria and 0 otherwise; follows a Bernoulli probability distribution. Conditionally on a zero-mean stationary Gaussian process $S(l)$ and additional set of study location specific random effects b_j, the linear predictor of the model assumes the form:

$$log(\frac{p_{ij}}{1-p_{ij}}) = x'_{ij}\beta + S(l_j) + b_j. \tag{3}$$

In Eqn 3 x is a vector of child, household, and cluster-level covariates with associated regression coefficients β, $S = (S(l) : l \in R^2)$ is a Gaussian process with mean zero, and variance σ^2, and correlation function $\rho(u) = Corr(S(l, S(l'))$. Among different parametric families, such as exponential, Gaussian, spherical have been proposed for $\rho(u)$. [41] recommends the use of Matern correlation function ([42]) given by

$$\rho(u;\phi,\kappa) = \frac{2^{\kappa-1}\Gamma(\kappa)}{(u/\phi)^{\kappa}\kappa_{\kappa}(u/\phi)}, u > 0$$

where $\phi > 0$ is a scale parameter, $\kappa > 0$ is the shape parameter, $\kappa_\kappa(\cdot)$ is the modified Bessel function of the second kind of order $\kappa > 0$, and $u = ||l - l'||$ is the Euclidean distance between two locations.

In Eqn 3, location-specific random effects b_j were included in the model to account for unexplained non-spatial variation. These random effects are assumed to be independent normal distributed with zero mean and variance τ^2 (i.e. $b_j \sim N(0, \tau^2)$), with τ^2 as the nugget effect accounting for the non-spatial variation. The marginal distribution of the outcome variable in Eqn 3 is a multivariate Gaussian process with

mean vector $X\beta$ and covariance matrix $\Sigma(\theta)$ with diagonal elements $\sigma^2 + \tau^2$ and off-diagonal elements are $\sigma^2\rho(u)$, with u the distance between locations l and l'.

Since Monte Carlo methods enable flexibility in fitting complex models and minimize computational problems encountered in the solely likelihood-based fitting ([43, 44]), in this study the model fitting was carried out using Monte Carlo maximum likelihood, as opposed to MCMC methods by considering the PrevMap package in R ([45]). The likelihood function for parameters β and $\theta^T = (\sigma^2, \phi, \tau^2)$ is obtained by integrating out the random effects included in Eqn 3, where σ^2 is the variance, ϕ is the range, and τ^2 is the nugget effect.

Spatial Prediction

For mapping, we predicted prevalence of infection at 7892 grid locations covering the entire Mozambique. Since it is difficult to get individual-level data at prediction location, The predictive map of malaria risk in Mozambique was created using the null geostatistical model 3.

Spatial distribution maps of malaria prevalence by survey clusters and regions of the country, and likelihood-based geostatistical modeling and spatial prediction were developed using R ([46]).

3 Results

A total of 4,347 children of age 6-59 months were tested for malaria from 221 nationally representative survey clusters. Table 1 presents the overall weighted proportion of children age 6-59 months classified as having malaria based on rapid diagnostic test(RDT) results according to different background characteristics. The mean age of children in this study was 32 months with standard deviation 15.5 month, and 64.57% of the children lived in rural areas. The overall weighted prevalence of malaria by RDT in Mozambique was 38.9% (with 95% CI: 36.9% - 40.8%).

According to their place of residence, the prevalence of malaria was 46.4% (with 95% CI: 43.9% - 48.9%) in rural areas, and 18.5% (95% CI:16.2%-21.1%) in urban areas (Table 1). Figure 1 (right panel) presents the contemporary malaria situation in survey locations of the country and can be used for malaria interventions in Mozambique.

Fig 1. Study clusters of the 2018 MMIS (left), and cluster level RDT prevalence (right panel)

Compared with the neighboring countries, the prevalence of malaria in Mozambique was more than twofold higher (Table 2).

The prevalence of malaria varies from province to province: lowest in Maputo (1%) and higher in Cabo Delgadi (57.3%) provinces of the country (Table 1, Fig 2, left panel).

Fig 2. Observed malaria prevalence at different provinces of Mozambique (left panel) and country altitude (right panel).

The results of geostatistical model which took into account the spatial correlation and non-spatial multilevel analysis by including survey design weights are given in Table 3.

From the geostatistical model, place of residence, mothers educational level, child age, household wealth index, child anaemia level, cluster-level malaria incidence rate and ITN-coverage were found significantly associated with malaria infection. In the geostatistical modeling part an exponential correlation function was assumed. The

Table 1. RDT prevalence of children of age 6-59 months classified as having malaria by different background characteristics and relative change between 2011 DHS and 2018 MMIS

Factors	2011 DHS			2018 MIS		
	n	**Proportion**	**95% CI**	n	**Proportion**	**95% CI**
**Residence**						
Urban	1599	0.167	(0.146,0.191)	1540	0.185	(0.162, 0.211)
Rural	3317	0.537	(0.442,0.483)	2807	0.464	(0.439, 0.489)
**Province**						
Niassa	434	0.521	(0.426,0.531)	446	0.483	(0.427, 0.539)
Cabo Delgado	413	0.472	(0.476,0.579)	398	0.573	(0.519, 0.625)
Nampula	411	0.433	(0.512,0.620)	434	0.478	(0.428, 0.529)
Zambezia	594	0.548	(0.409, 0.495)	457	0.445	(0.387, 0.505)
Tete	455	0.364	(0.583,0.684)	373	0.295	(0.245, 0.351)
Manica	480	0.279	(0.676,0.760)	510	0.473	(0.424, 0.522)
Sofala	645	0.305	(0.655,0.732)	487	0.293	(0.254, 0.336)
Inhambane	342	0.365	(0.578,0.689)	352	0.351	(0.300, 0.405)
Gaza	404	0.215	(0.740,0.823)	374	0.169	(0.129, 0.217)
Maputo Province	388	0.032	(0.944,0.982)	297	0.012	(0.004, 0.035)
Maputo City	350	0.0145	(0.965,0.994)	219	0.009	(0.002, 0.036)
**Mother education ~~label~~**						
No education	1538	0.467	(0.437,0.496)	908.00	0.52	(0.483, 0.562)
Primary	2261	0.383	(0.359,0.408)	1960	0.423	(0.395, 0.452)
Secondary/higher	1117	0.228	(0.199,0.261)	858	0.152	(0.114, 0.199)
**Child age(years**						
1	565	0.25	(0.208,0.297)	514	0.33	(0.277, 0.388)
2	1156	0.396	(0.362,0.431)	923	0.367	(0.326, 0.411)
3	1076	0.368	(0.334,0.403)	1000	0.429	(0.391, 0.470)
4	1089	0.416	(0.381,0.451)	933	0.379	(0.338, 0.422)
5	1030	0.414	(0.378,0.451)	977	0.414	(0.373, 0.456)
**Child sex**						
Male	2406	0.394	(0.371,0.417)	2154	0.397	(0.368, 0.425)
Female	2510	0.368	(0.346,0.392)	2193	0.381	(0.354, 0.408)
**HH Mosquito bednet**						
No	1751	0.403	(0.375,0.431)	388	0.483	(0.421, 0.620)
Yes	3165	0.369	(0.348,0.389)	3959	0.379	(0.359, 0.399)
**Wealth index**						
Poorest	874	0.548	(0.510,0.586)	852	0.58	(0.539, 0.620)
Poor	937	0.515	(0.478,0.553)	865	0.517	(0.476, 0.559)
Middle	955	0.412	(0.376,0.450)	815	0.422	(0.375, 0.469)
Rich	1100	0.257	(0.227,0.290)	988	0.207	(0.177, 0.241)
Richest	1050	0.054	(0.040,0.074)	827	0.028	(0.017, 0.047)
**Anaemia level**						
Non-anaemic	1699	0.478	(0.148,0.193)	974	0.218	(0.181, 0.259)
Anaemic	3217	0.169	(0.457,0.498)	3369	0.434	(0.412, 0.456)
**Total/National Level**	**4916**	**0.381**	**(0.365,0.397)**	4347	0.389	(0.369,0.408)

Table 2. Prevalence of malaria in children under five years from recent surveys in neighboring countries of Mozambique

Neighboring country	Survey year & data source	Child malaria prevalence(%)
Tanzania	MIS 2017	7.312
Malawi	MIS 2017	15.203
Zambia	MIS 2018	9.011
Zimbabwe	MIS 2016	0.202

assumption proved to be correct as the correlation function is supported by the empirical variogram (Figure 3).

Fig 3. Plots from variogram diagnostic check for the presence of residual spatial correlation (left-hand panel) and for compatibility of the data with the fitted geostatistical model 3 (right-hand panel). The solid line is the empirical variogram of the data. The shaded areas are 95% tolerance bands under the hypothesis of spatial independence (left-hand panel) and under the fitted model 3, (right-hand panel).

For a child living in a rural area, the odds of being malaria parasitaemia is 2.9 times as large as the odds of a child living in urban areas. The odds of being malaria-positive for anaemic child is 3.5 times that of non-anaemic child. Further, the odds of being malaria-positive for a child whose mother education level is secondary or higher is 0.64 times less likely compared with a child whose mothers are not educated.

The likelihood of being parasitaemia for children living with middle and better wealth index household were less likely compared with children living with poor(est) wealth index. Child sex and the availability of bed nets in the household were not significantly related with malaria prevalence ($P - value > 0.05$).

Compared with the non-spatial variation ($\tau^2 = 0.508$) the spatial variation is higher ($\sigma^2 = 0.697$). In the standard non-spatial multilevel modeling, the variance of the random intercept which corresponds to the cluster-level variability is 0.844. Using the non-spatial model the intraclass correlation is 0.204, ($\frac{0.844}{0.844+3.29} = 0.204$), implying that two subjects located in the same cluster had a correlation equal to 0.204 to be parasitaemia.

Figure 4 presents spatial predictions over the study locations, fixing the model parameters at the MCML estimates without any covariate. It also provides the predictive distribution of prevalence in each grid cell through the marginal prevalence (left panel) and probability that the estimated prevalence is above 20% (right panel). Areas with greater than 80% probability of exceeding the threshold were considered hot spots. Central and Northern provinces of Mozambique have locations with predicted prevalence above 20%. The dark green areas show locations where prevalence is above 20%, at 80% certainty.

Fig 4. Malaria prevalence predictions among children aged under five year in Mozambique (left panel) and exceedance probabilities (right panel) for the MMIS data.

Table 3. Parameter estimates from the geostatistical model (3) of malaria prevalence in children under five years of age in Mozambique

**Factors**	AOR	95% AOR CI
$\hat{\beta}_0$	0.366	(0.195,0.684)
Child level factors		
Age (in moth)	1.016	(1.010,1.021)
**Sleep under bed net** (ref: No)	0.736	(0.557,0.971)
**Anemia** (ref:not anemic)	3.502	(2.809,4.367)
Household level factors		
**Wealth index**(ref:Poorest)		
Poor	0.999	(0.801,1.246)
Middle	0.674	(0.534,0.852)
Rich	0.523	(0.396,0.691)
Richest	0.186	(0.111,0.313)
**Mother education** (ref:No education)		
Primary	0.868	(0.712,1.053)
Secondary/higher	0.635	(0.455,0.887)
Cluster level factors		
**Residence** (ref:urban)		
Rural	2.902	(2.316,3.636)
**Province**(ref:Cabo Delgado)		
Niassa	0.743	(0.506,1.090)
Nampula	1.011	(0.714,1.429)
Zamboza	0.808	(0.579,1.130)
Tete	0.373	(0.243,0.572)
Manica	0.833	(0.587,1.182)
Sofala	0.605	(0.421,0.870)
Inhambane	1.277	(0.862,1.891)
Gaza	0.138	(0.084,0.227)
Maputo Province	0.021	(0.007,0.060)
Maputo City	0.032	(0.007,0.143)
**ITN coverage**	0.25	(0.110,0.567)
**Malaria incidence**	4.005	(1.765,9.088)
**Spatial Covariance Prams**		
σ^2	0.697	0.396
ϕ	0.803	0.564
τ^2	0.508	1.164

AOR stands for adjusted odds ratio, and CI for confidence interval.

4 Discussion

This study undertook geostatistical analysis of the 2018 MMIS data to identify determinant factors of malaria risk. It also produced contemporary malaria risk maps of Mozambique for children under five years of age across survey clusters and regions of Mozambique. The generated spatially referenced malaria risk map is the first of its kind for Mozambique from a nationally representative geographically-referenced malaria indicator survey data. The map produced illustrates an important synopsis of prevalence of malaria in the country. Therefore, the observed predicted maps can serve as a resourceful tool in planning interventions and a reference point in evaluating their impact across different administrative regions of the country. The predicted map (Figure 4) show that, the health impact of malaria is higher in the northern and lowest in the southern part of the country. This Figure shows that most areas in the northern part of the country are well above a threshold of 20% prevalence. This implies that in these areas, control efforts towards malaria elimination can be considered. For malaria eradication programme implementers, control efforts in these areas would be on reducing transmission through preventive interventions such as mass bed-net distribution and/or indoor residual spraying campaigns. Thus, in the identified high transmission areas, control efforts would need to be more targeted and tailor-made as opposed to universal coverage effort, in order to cut transmission as much as possible. On the other hand, a number of localities in southern part of the country have prevalence below 20% which require less resource to eradicate malaria s compared with the northern part of the country. The following regions: Tete, Gaza, Maputo and Maputo city have locations where predicted malaria prevalence in children under 5 years is less than 20%.

Malaria risk maps generated by ([4, 25]) relied on historical survey data and do not reflect current malaria prevalence situation for under-five year children in Mozambique. Further, the analyses done by ([5, 7, 22, 23]) focus on mapping the prevalence of malaria in a certain specific province which does not represent national burden of malaria risk in children under-five years of age. The study conducted by ([48]) does not represent the contemporary situation of malaria in Mozambique.

As presented in other similar studies conducted to analyze malaria indicator survey datasets ([14, 15, 18, 19]) the likelihood of having malaria increased as a child gets older. This positive relationship between malaria and child age showed that, the older the child the higher chance to be infected by malaria. This may be due to declining breast feeding which exposes a child to maternal antibodies.

We observed that prevalence estimates vary across different socio-demographic groups as well as different regions of the country. The highest prevalence was observed in Cabo Delgado (57.3% with 95% CI: 51.9-62.5%), Niassa (48.3% with 95% CI: 42.7-53.9%), and Nampula (47.8% with 95% CI: 42.8-52.9%) provinces of the country. This may be due to the fact that, the most populous provinces Zambezia and Nampula have the worst education and health outcomes; and in general northern provinces have worse infrastructure; higher levels of environmental degradation and less economic activity than the south [49]. Further, a secondary school access is unevenly distributed among the provinces and lowers in Cabo Delgado and Niassa province [50].

The lowest prevalence was observed in Maputo province and Maputo city (Table 1). Children living in urban areas had significantly lower risk of having malaria compared with children in rural areas. Children who live in rural areas were 2.9 times more likely to have malaria than those who live in urban areas (adjusted odds ratio (AOR) = 2.902). Similar results were also found in other studies ([13, 18, 51]). Among many other factors, malaria plays a major causative role of anaemia globally ([11, 52–55]). In previous studies, the overall prevalence of anaemia in under-five children in Mozambique was above 70% ([48]). In this study, we found that anaemic children were found 3.5 times higher to be parasitaemia than non-anemic children.

The positive relationship with age indicated that the older the child the higher the risk of contacting malaria. This findings agrees with results reported from analyses of MIS data in Nigeria ([11]), Angola ([12]), Tanzania ([13]), Burkina Faso ([15]), The Gambia ([40]),Cote d'Ivoire ([51]), Malawi ([20]) and Uganda ([18]).

Recently, Amoah and his colleagues ([56]) studied the impact of malaria on child growth using 20 Demographic and Health Surveys conducted in 13 African countries. Their result reveals' that malaria had a significant negative effect on child growth.

In line with other similar studies ([11–13, 17, 18, 40]), our study findings showed that malaria prevalence is strongly associated with mothers' education level, child age, wealth index, cluster level malaria incidence rate, and place of residence. Household wealth index and mothers' educational level were negatively associated with the prevalence of malaria, suggesting that the higher the wealth index and the higher mothers' educational level the lower the risk of acquiring malaria. Supporting our findings, [12, 13] and [18] found a decreased risk for malaria among children living with better household wealth index, and higher mothers education.

The results presented in this study should be considered in light of some limitations. Since the analysis result in this study derived from a nationwide cross-sectional malaria indicator survey, sub-national variations in risk and epidemiological transitions could be triangulated with additional routine data from health information systems and malaria hospitalization. Further, employing model-based geostatistical methods to interpolate information on malaria prevalence at province/locality levels is less perfect when compared to complete, reliable routine data on the monthly presentation of parasitologically diagnosed fevers to health facilities. Since malaria is environmentally mediated infectious disease, in future studies, considering environmental factors [21, 57] in the covariance structure of the model will yield better prediction map.

5 Conclusion

Among other health problems, malaria remains one of the biggest public health problems in Mozambique. Thus, evidence-based interventions are needed to reduce the economic burden ([58–60]), and malaria related diseases in the country. In this respect, the results of this study are useful to make geographically targeted interventions.

The results of study showed that high spatial variation in malaria risk were observed across provinces with higher prevalence in the northern part and lower in the southern part of the country. Children living in urban areas had the lowest risk of infection compared with children living in rural areas indicating that more efforts is needed in those areas. Furthermore our result revealed that malaria risk is linked with child age, household wealth index, mother's educational level, place of residence and child's anaemia level. Moreover, household level determinants of malaria infection that are identified by malaria prevalence maps at cluster and province level could be used in malaria control implementing programs to identify priority intervention areas.

Acknowledgment

The author thanks Professor Eshetu Wencheko, Dr. Birhanu Teshome, and anonymous reviewers for their valuable comments. The paper has been considerably strengthened by their comments.

References

1. WHO. Tailoring malaria interventions in the COVID-19 response; 2020.

2. WHO. Malaria eradication: benefits, future scenarios & feasibility: A report of the Strategic Advisory Group on Malaria Eradication.; 2020.

3. WHO. World malaria report 2018; 2018.

4. Bhatt S, Weiss D, Cameron E, Bisanzio D, Mappin B, Dalrymple U, et al. The effect of malaria control on Plasmodium falciparum in Africa between 2000 and 2015. Nature. 2015;526(7572):207–211.

5. Zacarias O, Andersson M. Spatial and temporal patterns of malaria incidence in Mozambique. Malaria Journal. 2011;10(189).

6. PMI. President's Malaria Initiative (PMI): Malaria Operational Plan FY 2019; 2019.

7. Ferrao J, Niquisse S, Mendes PM JM. Mapping and Modelling Malaria Risk Areas Using Climate, Socio-Demographic and Clinical Variables in Chimoio, Mozambique. International Journal of Environmental Research and Public Health. 2018;15(795).

8. Nyarko S, Cobblah A. Socio-demographic determinants of malaria among under-five children in Ghana. Malar Res Treat. 2014;.

9. Afoakwah C, Deng X, Onur I. Malaria infection among children under five: the use of large-scale interventions in Ghana. BMC Public Health. 2018;18(536).

10. Roberts D, Matthews G. Risk factors of malaria in children under the age of five years old in Uganda. Malaria Journal. 2016;15(246).

11. Adigun AB, Gajere EN, Oresanya O, Vounatsou P. Malaria risk in Nigeria: Bayesian geostatistical modelling of 2010 malaria indicator survey data. Malaria Journal. 2015;14(156).

12. Gosoniu L, Veta AM, Vounatsou P. Bayesian Geostatistical Modeling of Malaria Indicator Survey Data in Angola. PLOS ONE. 2010;5(3).

13. Gosoniu L, Msengwa A, Lengeler C, Vounatsou P. Spatially explicit burden estimates of malaria in Tanzania: bayesian geostatistical modeling of the malaria indicator survey data. PloS One. 2012;7(5).

14. Riedel N, Vounatsou P, Miller JM, Gosoniu L, Chizema-Kawesha E, Mukonka V, et al. Geographical patterns and predictors of malaria risk in Zambia: Bayesian geostatistical modelling of the 2006 Zambia national malaria indicator survey (ZMIS). Malaria Journal. 2010;9(37).

15. Samadoulougou S, Maheu-Giroux M, Kirakoya-Samadoulougou F, De Keukeleire M, Castro M, Robert A. Multilevel and geo-statistical modeling of malaria risk in children of Burkina Faso. Parasites & Vectors. 2014;7(1):350.

16. Khagayi S, Amek N, Bigogo G, Odhiambo F, Vounatsou P. Bayesian spatio-temporal modeling of mortality in relation to malaria incidence in Western Kenya. PLoS ONE. 2014;12(17):e0180516.

17. Kazembe LN, Kleinschmidt I, Holtz TH, Sharp BL. Spatial analysis and mapping of malaria risk in Malawi using point-referenced prevalence of infection data. International Journal of Health Geographics. 2006;5(41).

18. Ssempiira J, Nambuusi B, Kissa J, Agaba B, Makumbi F, Kasasa S, et al. Geostatistical modelling of malaria indicator survey data to assess the effects of interventions on the geographical distribution of malaria prevalence in children less than 5 years in Uganda. PLoS ONE. 2017;12(4:):e0174948.

19. Yankson F, Anto E, Chipeta M. Geostatistical analysis and mapping of malaria risk in children under 5 using point-referenced prevalence data in Ghana. Malaria Journal. 2019;18(67).

20. Chipeta M, Giorgi E, Mategula D, et al. Geostatistical analysis of Malawi's changing malaria transmission from 2010 to 2017. Wellcome Open Research. 2019;4(57).

21. Ejigu B, Wencheko E, Moraga P, Giorgi E. Geostatistical methods for modelling non-stationary patterns in disease risk. Spatial Statistics. 2020;35(100397).

22. Zacarias O, Majlender P. Comparison of infant malaria incidence in districts of Maputo province, Mozambique. Malaria Journal. 2011;10(93).

23. Giardina F, Jonas Franke J, Vounatsou P. Geostatistical modelling of the malaria risk in Mozambique: effect of the spatial resolution when using remotely-sensed imagery. Geospatial Health. 2015;10(333).

24. Hay SI, Snow RW. The Malaria Atlas Project: Developing Global Maps of Malaria Risk. PLoS Med. 2006;3(12):e473.

25. Gething PW, Patil AP, Smith DL, Guerra CA, Elyazar IR, Johnston GL, et al. A new world malaria map: Plasmodium falciparum endemicity in 2010. Malaria Journal. 2011;10(378).

26. Instituto, Nacional, de Saúde. Inquérito Nacional sobre Indicadores de Malária em Moçambique 2018; 2019.

27. Benjamin M, Fish TD, Eitelberg D, Dontamsetti T. The DHS Program Geospatial Covariate Datasets Manual (Second Edition); 2018.

28. da Saude M. Moçambique Inquérito Demográfico e de Saúde 2011; 2011. Available from: http://dhsprogram.com/pubs/pdf/FR266/FR266.pdf.

29. Carle AC. Fitting multilevel models in complex survey data with design weights: recommendations. BMC Medical Research Methodology. 2009;9:49–62.

30. Agresti A, Coull BA. Approximate is better than 'exact' for interval estimation of binomial proportions. The American Statistician. 1998;52:119–126.

31. Wilson E. Probable inference, the law of succession, and statistical inference. Journal of the American Statistical Association. 1927;22:209–212.

32. Kott PS, Carr DA. Developing an Estimation Strategy for a Pesticide Data Program. Journal of Official Statistics. 1997;13(4):367–383.

33. Franco C, Little RA, Louis TA, Slud EV. Comparative Study of Confidence Intervals for Proportions in Complex Sample surveys. Journal of Survey Statistics and Methodology. 2019;7:334–364.

34. Rabe-Hesketh S, Skrondal A. Multilevel modelling of complex survey data. Royal Statistical Society (Series A). 2006;169(4):805–827.

35. Asparouhov T. General Multi-Level Modeling with Sampling Weights. Communications in statistics -Theory and methods. 2006;35(3):93–103.

36. Grilli L, Pratesi M. Weighted estimation in multilevel ordinal and binary models in the presence of informative sampling designs. Survey Methodology. 2004;30:93–103.

37. Pfeffermann D. Modelling of complex survey data: why mode? Why is it a problem? How can we approach it? Survey Methodology. 2011;37:115–136.

38. Pfeffermann D, Skinner CJ, Holmes DJ, Goldstein H, Rasbash J. Weighting for unequal selection probabilities in multilevel models. Royal Stat Soc. 1998;60:23–40.

39. Diggle P, Tawn J, Moyeed R. Model-based geostatistics. Journal of the Royal Statistical Society Series C (Applied Statistics). 1998;47:299–350.

40. Diggle P, R M, Rowlingson B, Thomson M. Childhood Malaria in the Gambia: A Case-Study in Model-Based Geostatistics. Journal of the Royal Statistical Society Series C (Applied Statistics). 2002;51(4):493–506.

41. Stein M. Interpolation of Spatial Data: Some Theory for Kriging. New York: Springer; 1999.

42. Matern B. Spatial Variation. Berlin: Springer-Verlag; 1986.

43. Geyer CJ, Thompson EA. Constrained Monte Carlo Maximum Likelihood for Dependent Data. Journal of the Royal Statistical Society B. 1992;54(3):657–699.

44. Geyer CJ. On the Convergence of Monte Carlo Maximum Likelihood Calculations. Journal of the Royal Statistical Society B. 1994;56(1):261–274.

45. Giorgi E, Diggle PJ. PrevMap: an R package for prevalence mapping. Journal of Statistical Software. 2017;78(8):1–29.

46. R Core Team. R: A Language and Environment for Statistical Computing; 2018. Available from: https://www.R-project.org/.

47. StataCorp. Stata Statistical Software: Release 14; 2015. Available from: https://www.stata.com/.

48. Mabunda S, Casimiro S, Quinto L, Alonso P. A country-wide malaria survey in Mozambique. I. Plasmodium falciparum infection in children in different epidemiological settings. Malaria Journal. 2008;7(216).

49. SIDA. MOZAMBIQUE MULTIDIMENSIONAL POVERTY ANALYSISSTATUS AND TRENDS; 2019.

50. Fox L, Santibanez L, Nguyen N, Andre P. Education Reform in Mozambique: Lessons and Challenges; 2012.

51. Raso G, Schur N, Utzinger J, Koudou BG, Tchicaya ES, Rohner Fea. Mapping malaria risk among children in Cote d'Ivoire using Bayesian geo-statistical models. Malaria Journal. 2012;11(160).

52. Korenromp EL, Armstrong-Schellenberg JR, Williams BG, Nahlen BL, Snow RW. Impact of malaria control on childhood anaemia in Africa: a quantitative review. Trop Med Int Health. 2004;9(10):1050–1065.

53. Naing C, Sandhu NK, Wai VN. The Effect of Malaria and HIV Co-Infection on Anemia: A Meta-Analysis. Medicine. 2016;95(14).

54. Kassebaum NJ, Jasrasaria R, M N, Wulf SK, Johns N. A systematic analysis of global anemia burden from 1990 to 2010. Blood. 2014;123(5):615–624.

55. Ejigu B, Wencheko E, Berhane K. Spatial pattern and determinants of anaemia in Ethiopia. PLoS ONE. 2018;13(5):e0197171.

56. Amoah B, Giorgi E, Heyes DJ, Burren S, Diggle P. Geostatistical modelling of the association between malaria and child growth in Africa. International Journal of Health Geographics. 2017;17(7).

57. Ejigu B, Wencheko E. Introducing Covariate Dependent Weighting Matrices in Fitting Autoregressive Models and Measuring Environmental Autocorrelation. Spatial Statistics. 2020;38(100454).

58. Aryeetey GC, Agyemang SA, Aubyn VN, Aikins M, Bart-Plange CN, Malm KL, et al. Economic burden of malaria on businesses in Ghana: a case for private sector investment in malaria control. Malaria Journal. 2016;15(454).

59. Gallup JL, Sachs JD. The economic burden of malaria. Am J Trop Med Hyg. 2001;64(Suppl 1):85–96.

60. Sicuri E, Vieta A, Lindner L, Constenla D, Sauboin C. The economic costs of malaria in children in three sub -Saharan countries: Ghana, Tanzania and Kenya. Malaria Journal. 2013;12(307).

Figure 1

Click here to access/download;Figure;Fig 1.png

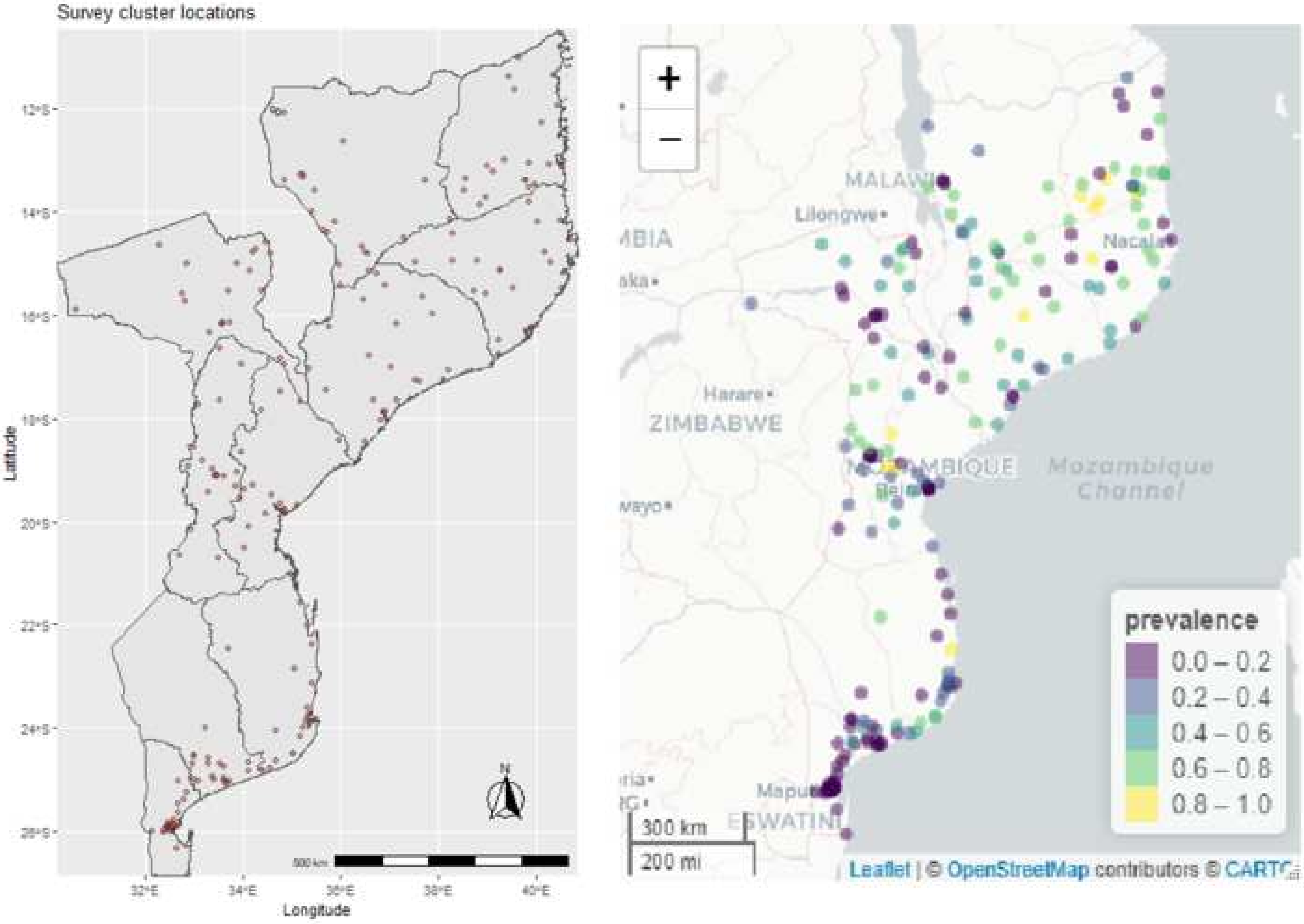

Figure 2

Click here to access/download;Figure;Fig 2.png

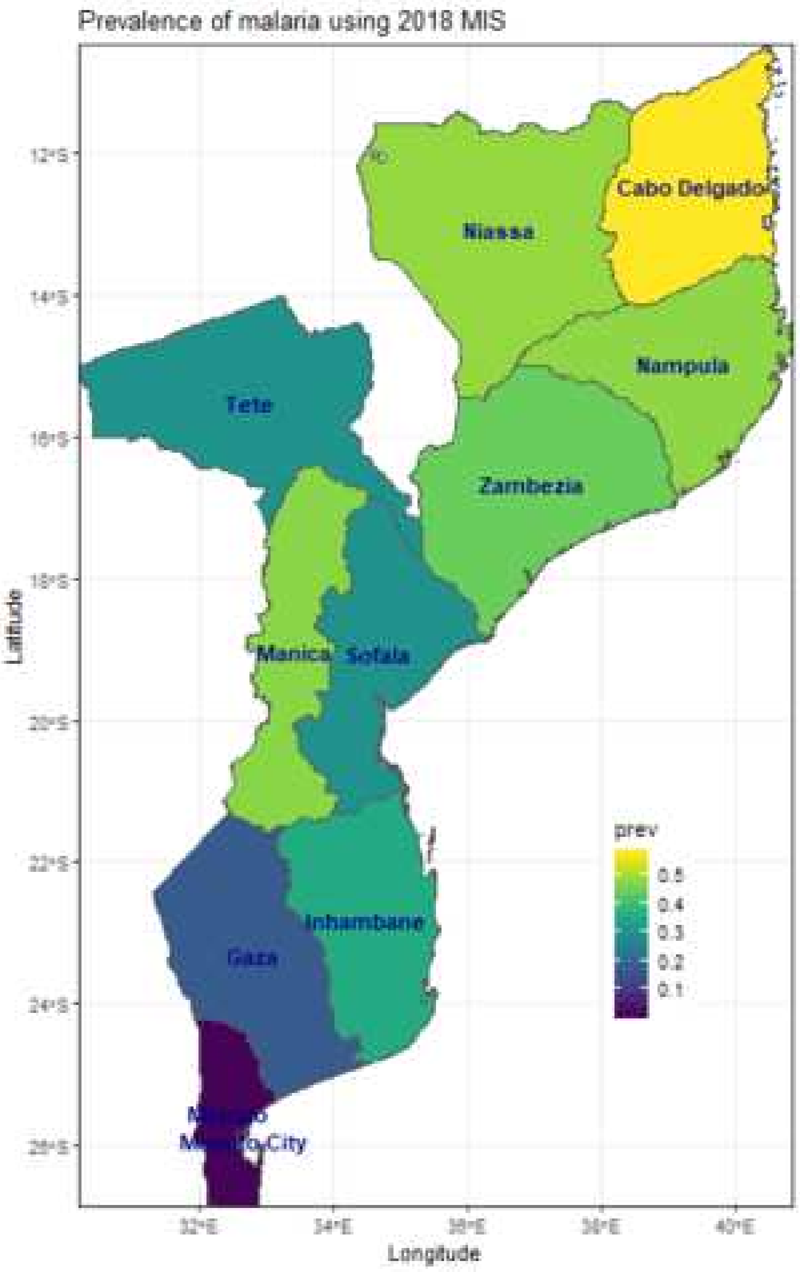

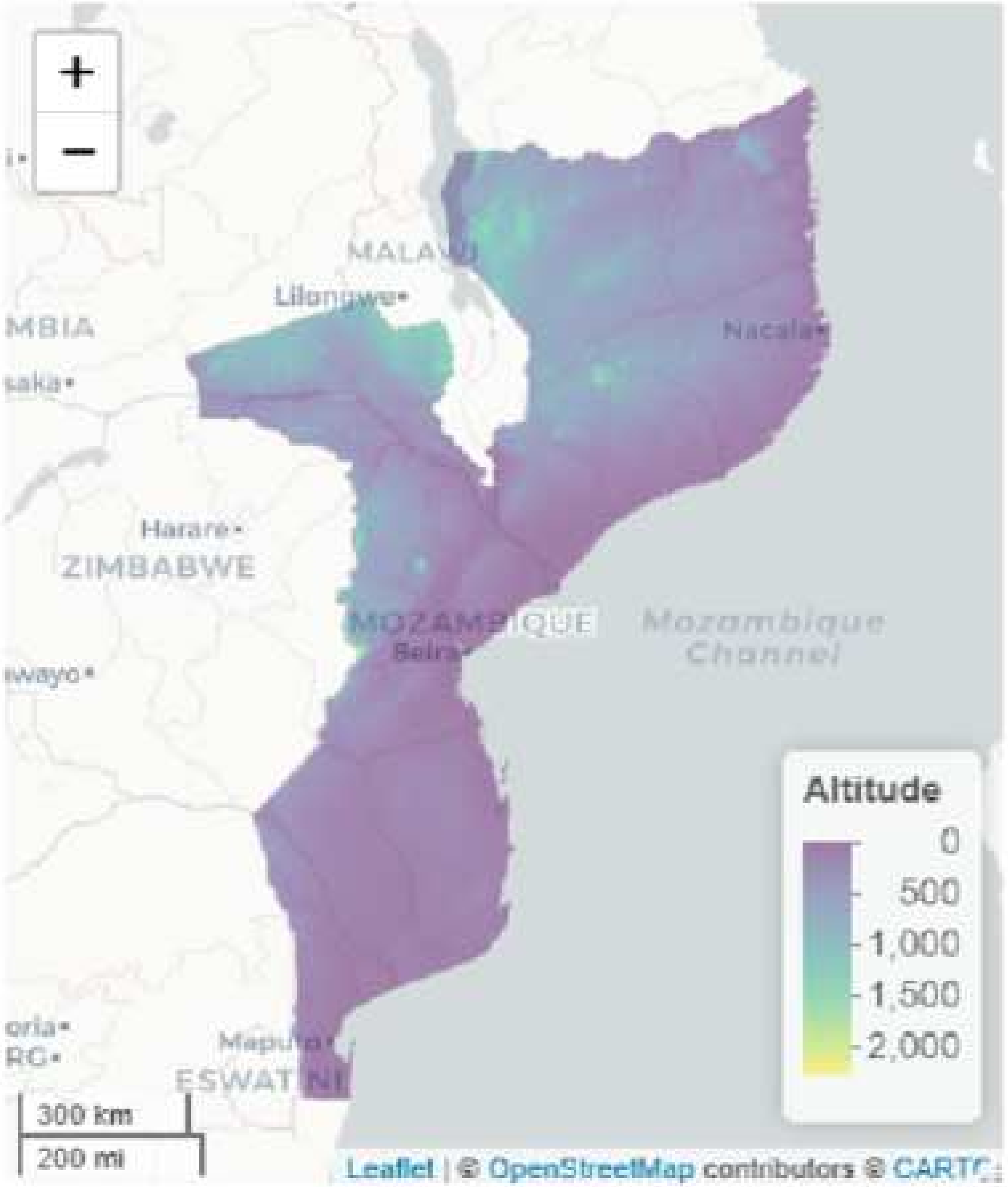

Figure 3

Click here to access/download;Figure;Fig 3.png

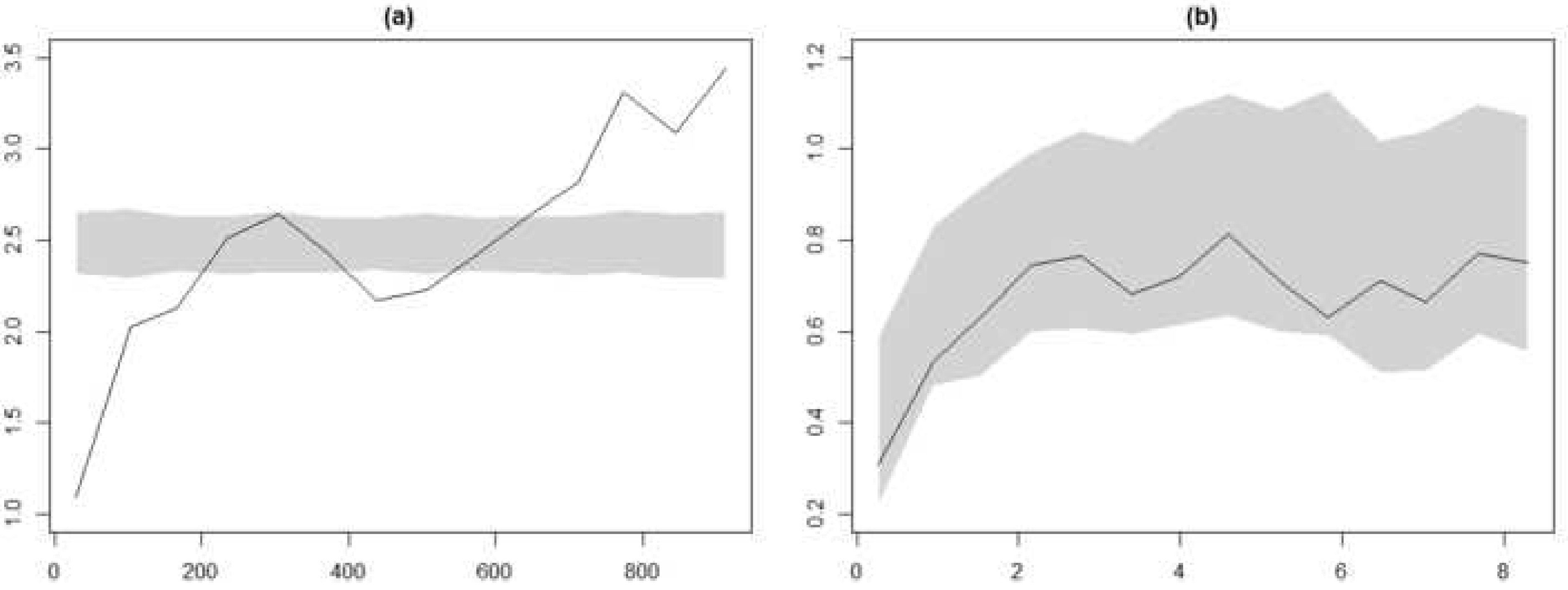

Figure 4

Click here to access/download;Figure;Fig 4.png

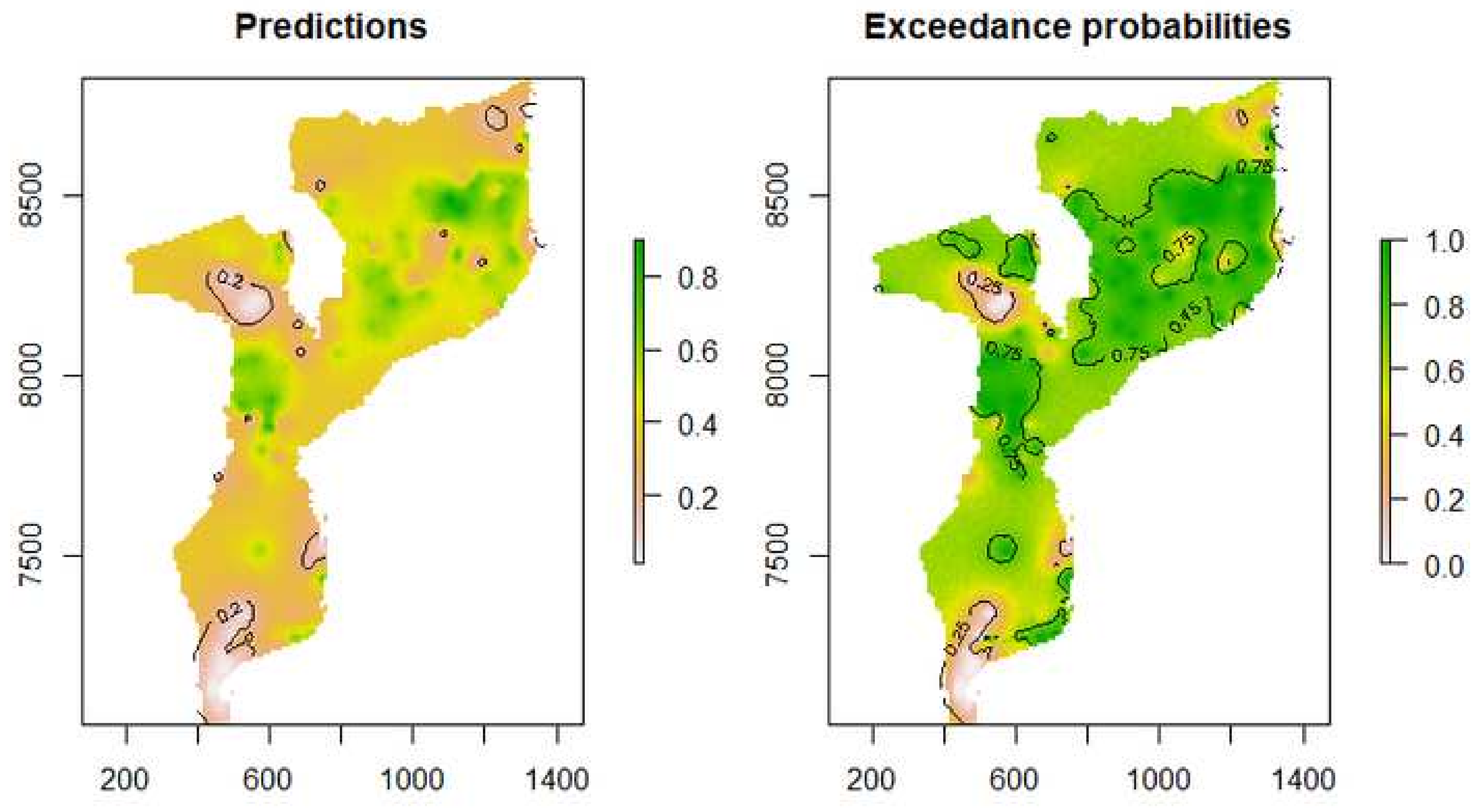

Geostatistical Analysis and Mapping of Malaria Risk in Children of Mozambique

Bedilu Alamirie Ejigu[1*],

1 Department of Statistics, College of Natural and Computational Sciences, Addis Ababa University, Addis Ababa, Ethiopia.

* Corresponding author
E-mail: bedilu.alamirie@aau.edu.et (B.A Ejigu)

abstract

Malaria remains one of the most prevalent infectious diseases in the tropics and subtropics, and Mozambique is not an exception. To design geographically targeted and effective intervention mechanisms of malaria, an up-to-date map that shows the spatial distribution of malaria is needed. This study analyzed 2018 Mozambique Malaria Indicator Survey using geostatistical methods to: i) explore individual, household, and community-level determinants of malaria in under-five children, ii) prepare a malaria prevalence map in Mozambique, and iii) produce prediction prevalence maps and exceedence probability across the country. The results show the overall weighted prevalence of malaria was 38.9% (N=4347, with 95% CI: 36.9% - 40.8%). Across different provinces of Mozambique, the prevalence of malaria ranges from 1% in Maputo city to 57.3% in Cabo Delgado province. Malaria prevalence was found to be higher in rural areas, increased with child's age, and decreased with household wealth index and mother's level of education. Given the high prevalence of childhood malaria observed in Mozambique there is an urgent need for effective public health interventions in malaria hot spot areas. The household determinants of malaria infection that are identified in this study as well as the maps of parasitaemia risk could be used by malaria control program implementers to define priority intervention areas.

1 Background

Malaria is an infectious disease caused by a parasite that is transmitted from one subject to another by blood-sucking female anopheles mosquitoes. It is a major public health problem, especially in Africa and Asia. Many countries made an incredible progress in the fight against malaria, and as a result malaria deaths have fallen by more than 50% globally between 2000 and 2015. Seventeen countries eliminated malaria, and six were certified by WHO as malaria-free ([1]). Based on WHO recent report, on a global scale, progress has levelled off but no gains were achieved in reducing malaria case incidence over the last five years ([2]). The WHO strategic advisory group predicts that there will be 11 million cases of malaria in Africa by 2050 ([2]). The current COVID-19 pandemic places extra burden on health systems worldwide, and especially in sub-Saharan Africa which accounts for more than 90% of global malaria cases and deaths. Recently, WHO recommends, countries to move quickly to save lives from malaria in sub-Saharan Africa ([1]).

In Africa, more than two-thirds of all malaria deaths occur in children under-five years old ([3]). Because of a continual fight against malaria by intervention programs,

malaria infection prevalence and clinical incidence decreased by 50% and 40%, between 2000 and 2015, respectively ([4]). In 2017, an estimated 219 million cases and 435 thousand deaths of malaria occurred worldwide, of which 200 million (92%) malaria cases were in the WHO African Region ([3]). Fifteen sub-Saharan Africa countries and India carried 80% of the global malaria burden and Mozambique accounts for 5%. Children under 5 years of age are the most vulnerable group affected by malaria and they accounted for 61% of all malaria deaths worldwide ([3]).

In Mozambique, malaria is a common disease with seasonal fluctuation in all parts of the country with seasonal peak ranging from December to April ([5]). The prevalence varies across different ecological zones and transmission occurs year-round with relatively higher prevalence in the northern part of the country. Various factors influence the dynamics of malaria transmission and infection ranging from natural (i.e. rainfall, temperature) to social factors. Previously conducted national surveys showed that the prevalence of malaria in under five children was 39% and 40% in the years 2011 and 2015, respectively. Malaria accounts for 29% of all deaths and 42% of deaths in under-five children in the country ([6]).

Even though, Mozambique's entire population is at risk of malaria due to different environmental and ecological factors ([7]), pregnant women and under-five children are at higher risk of severe illness due to their low immunity ([8–10]). Thus, efficient interventions and preventive measures could be improved by advancing our understanding of the spatial patterns of malaria prevalence distribution and the underlying factors. Long-lasting insecticidal nets (LLINs) is one of the main interventions mechanisms for preventing malaria infection, and the 2017-2022 Mozambique strategic plan aims to achieve one net for every two people LLINs coverage across the country ([6]). Currently, disruptions to insecticide-treated net campaigns due to COVID-19 pandemic and in access to antimalarial medicines could lead to ~~a doubling~~ increase in the number of malaria deaths and co-morbidities. This requires malaria affected countries like Mozambique ~~could~~ should identify malaria hot-spot areas and move quickly to save lives from malaria. The spatial distribution risk map of malaria is an important tool for effective planning, malaria control intervention, resource mobilization, monitoring and evaluation process. As a result, to advance intervention mechanisms, spatial distribution maps of malaria prevalence across the study area have been produced using geostatistical modeling approaches ([11–21]) with the aim of identifying areas where greatest control effort should be focused. Previously generated maps depicted the geographical distribution of malaria risk in Mozambique either at province or continental-scale ([4, 7, 22–25]), not at country-level. However, those maps may not reflect the current malaria situation in the country, as they rely on historical and outdated survey data. Malaria risk maps based on historical data cannot reflect the current situation which is changing due to ongoing interventions.

To date, to our knowledge, the only map available for the spatial distribution of malaria prevalence in Mozambique based on recent data was produced for Chimoio ([7]) and Maputo province ([5, 22]) which do not represent the current situation since it does not take into account contemporary effects of interventions and socio-economic status. Further, recently produced reports related with malaria in Mozambique do not address the spatial distribution of malaria across survey clusters of the country by taking into account other factors ([6, 26]).

In this study, the 2018 Mozambique Malaria Indicator Survey (MMIS) data were analyzed using geostatistical modeling approaches to: i) identify determinant factors associated with malaria risk, ii) produce a prevalence map of malaria among children under the age of five across survey clusters and regions of Mozambique, and iii) produce prediction prevalence maps and exceedence probability across the country. Predicted malaria prevalence maps generated in this study would help policy makers to identify

high-risk areas and design targeted interventions.

2 Materials and Methods

2.1 Mozambique MIS Data

The data for this study were obtained from the 2018 Mozambique Malaria Indicator Survey (MMIS). The main aim of this survey was to obtain population-based estimates of malaria indicators by considering a nationally representative dataset which serves as input for strategic planning and evaluation of malaria control program([26]). Stratified two-stage sampling technique was used to select enumeration areas (EAs) and households. Sampling procedures of the survey have been mentioned in the survey final report ([26]). Permission to use the 2018 MMIS data was obtained from the DHS website (`www.dhsprogram.com`). Figure 1 (left panel) presents the map of survey cluster locations where raw dataset were collected, and prevalence is depicted (right panel).

The geostatistical modeling includes: individual-level variables:child age, gender, anaemia level, child slept under bed net; household-level variables including educational level of the mother, household wealth index, and availability of bed nets in the household; and community-level variables: place of residence, mean temperature, estimated malaria incidence in the cluster, ITN coverage, and region were used in the analysis. Cluster-level geospatial data used in this study (ITN-coverage, malaria incidence) were obtained from DHS Program Geospatial Covariate Datasets (`www.dhsprogram.com`), and the construction procedures of geospatial data were explained in [27]. Further, to see the change in the prevalence of malaria by different factors in the past seven years, the 2011 Mozambique demographic and health survey used [28]. In addition to this, to compare the overall risk of malaria with neighboring countries, nationwide malaria prevalence in Tanzania, Malawi, Zambia, and Zimbabwe considered.

2.2 Statistical Analysis

Malaria indicator survey data-sets are often complex in nature for two reasons: i) the use of stratified multistage cluster sampling to increase sampling and cost efficiency, and ii) unequal probabilities of selection from target-populations for sampled elements, often as a result of oversampling of key subgroups. Thus, our data analysis tools employed sampling weights for generating unbiased population estimates ([29]).

2.2.1 Weighted Confidence Interval for Proportions

Constructing a confidence interval for proportion p is one of the most basic analyses in statistical inference, and it is an important aspect of reporting statistical results. Let Y denote a binomial variate for sample size n, and let $\hat{p} = \frac{Y}{n}$ denote the sample proportion. Under asymptotic normality of the sample proportion and estimating the standard error, an approximate $100(1-\alpha)\%$ confidence interval for P is

$$\hat{p} \mp z_{1-\alpha/2}\sqrt{\hat{p}(1-\hat{p})/n}. \quad (1)$$

The large-sample Wald intervals (Eqn 1) are known to perform poorly ([30]), but the Wilson intervals ([31]) given by Eqn 2 have been shown to perform well in a variety of situations.

$$\frac{\hat{p} + z^2_{1-\alpha/2}/2n}{1 + z^2_{1-\alpha/2}/n} \mp \frac{z_{1-\alpha/2}\sqrt{\hat{p}(1-\hat{p}) + z^2_{1-\alpha/2}/4n}}{\sqrt{n}(1 + z^2_{1-\alpha/2}/n)} \quad (2)$$

Both Wald (Eqn 1) and Wilson (Eqn 2) intervals are appropriate for survey data with simple random sampling designs, but they are not designed to accommodate clustering or unequal weights of more complex sample surveys, like the data analyzed in this manuscript.

A common approach to construct confidence intervals for proportions from complex sample survey data is to modify the inputs of Eqn 2 to account for survey weights and the design effect. The survey-weighted, estimated proportion,$\hat{p}$, is used along with a consistent design-based estimate, $\hat{Var}(\hat{p})$, of its variance. For complex survey data. [32] propose a modified version by replacing $z_{1-\alpha/2}$ with $t_c(1-\alpha/2)$ in equation Eqn 2, and replacing n with the effective sample size, defined as $n_{eff} = n/\hat{Deff}(\hat{p})$ where

$$\hat{Deff}(\hat{p}) = \frac{\hat{Var}(\hat{p})}{\hat{p}(1-\hat{p})/n} = \frac{\sum_h (\frac{N_h}{N})^2 (1-\frac{n_h}{N}) \frac{\hat{p}(1-\hat{p})}{n_h}}{\hat{p}(1-\hat{p})/n} = \frac{n\sum_i^n w_i^2}{(\sum_i^n w_i)^2}$$

as the estimated design effect ([33]), w_i is the weight of the ith unit selected in the sample, w represents sampling weights that denote the inverse of the probability that the observation is included because of the sampling design.

2.2.2 Weighted Multilevel Modeling

~~Multilevel modeling has become a common way of model fitting procedure in large scale assessment surveys with multistage sampling like MMIS and unequal probability of selection ([29, 34]). Failing to account for this aspect of the design can lead to biased parameter estimates ([35–37]). To address this problem, analysts have recommended incorporating design weights in the likelihood function ([29, 36–38]). [38] developed a method for applying sampling weights to multilevel samples by defining a pseudolikelihood using the following procedure.~~

> ~~Partition the weights by various levels of the model; Normalize the weights at each level; and Apply normalized weights to each level of the multilevel likelihood to create a pseudo likelihood.~~

~~In order to identify different risk factors, by taking in to account survey design weights, the following non-spatial multilevel mixed model is fitted to the data.~~

$$log(\frac{p_{ij}}{1-p_{ij}}) = \beta_o + x'_{ij}\beta + b_j, \tag{3}$$

~~In this model equation β_0 is an intercept, β is an unknown slope parameter for individual-level predictors, and b_j are mutually independent Gaussian random effects used to capture within-cluster correlation. In standard multilevel models, b_j is usually assumed to be a normally distributed random intercept with mean 0 and variance σ_b^2. Random intercepts models, like (Eqn 3) enable researchers to accommodate correlations within higher-level units resulting from clustered study designs.~~

2.2.3 Geostatistical Modeling

Non-spatial modelling approaches ~~(described in section 2.2.2)~~ assume independence between study locations where the data collected inadvertently neglect potential spatial dependency between neighboring locations due to unobserved common exposures. To overcome such limitations, geostatistical models relate disease prevalence data with potential predictors and quantify spatial dependence via the covariance matrix of a Gaussian process facilitated by adding random effects at the observed locations ([39]). Such type of geostatistical models have already been applied to model malaria risk at different geographical scales in different Africa countries ([11–13, 15, 19, 20, 40]).

In model equation 4 below, malaria status Y_{ij} of child i at location j take a value of 1 if the child has malaria and 0 otherwise; follows a Bernoulli probability distribution. Conditionally on a zero-mean stationary Gaussian process $S(l)$ and additional set of study location specific random effects b_j, the linear predictor of the model assumes the form:

$$log(\frac{p_{ij}}{1-p_{ij}}) = x'_{ij}\beta + S(l_j) + b_j. \quad (4)$$

In Eqn 4 x is a vector of child, household, and cluster-level covariates with associated regression coefficients β, $S = (S(l) : l \in R^2)$ is a Gaussian process with mean zero, and variance σ^2, and correlation function $\rho(u) = Corr(S(l, S(l'))$. Among different parametric families, such as exponential, Gaussian, spherical have been proposed for $\rho(u)$. [41] recommends the use of Matern correlation function ([42]) given by

$$\rho(u; \phi, \kappa) = \frac{2^{\kappa-1}\Gamma(\kappa)}{(u/\phi)^{\kappa}\kappa_{\kappa}(u/\phi)}, u > 0$$

where $\phi > 0$ is a scale parameter, $\kappa > 0$ is the shape parameter, $\kappa_{\kappa}(\cdot)$ is the modified Bessel function of the second kind of order $\kappa > 0$, and $u = ||l - l'||$ is the Euclidean distance between two locations.

In Eqn 4, location-specific random effects b_j were included in the model to account for unexplained non-spatial variation. These random effects are assumed to be independent normal distributed with zero mean and variance τ^2 (i.e. $b_j \sim N(0, \tau^2)$), with τ^2 as the nugget effect accounting for the non-spatial variation. The marginal distribution of the outcome variable in Eqn 4 is a multivariate Gaussian process with mean vector $X\beta$ and covariance matrix $\Sigma(\theta)$ with diagonal elements $\sigma^2 + \tau^2$ and off-diagonal elements are $\sigma^2\rho(u)$, with u the distance between locations l and l'.

Since Monte Carlo methods enable flexibility in fitting complex models and minimize computational problems encountered in the solely likelihood-based fitting ([43, 44]), in this study the model fitting was carried out using Monte Carlo maximum likelihood, as opposed to MCMC methods by considering the PrevMap package in R ([45]). The likelihood function for parameters β and $\theta^T = (\sigma^2, \phi, \tau^2)$ is obtained by integrating out the random effects included in Eqn 4, where σ^2 is the variance, ϕ is the range, and τ^2 is the nugget effect.

Spatial Prediction

For mapping, we predicted prevalence of infection at 7892 grid locations covering the entire Mozambique. Since it is difficult to get individual-level data at prediction location, The predictive map of malaria risk in Mozambique was created using the null geostatistical model 4.

Spatial distribution maps of malaria prevalence by survey clusters and regions of the country, and likelihood-based geostatistical modeling and spatial prediction were developed using R ([46]). ~~The weighted multilevel mixed model analyses which takes into account survey design weights was done using Stata ([47])~~.

3 Results

A total of 4,347 children of age 6-59 months were tested for malaria from 221 nationally representative survey clusters. Table 1 presents the overall weighted proportion of children age 6-59 months classified as having malaria based on rapid diagnostic test(RDT) results according to different background characteristics. The mean age of children in this study was 32 months with standard deviation 15.5 month, and 64.57% of the children lived in rural areas. The overall weighted prevalence of malaria by RDT in Mozambique was 38.9% (with 95% CI: 36.9% - 40.8%).

According to their place of residence, the prevalence of malaria was 46.4% (with 95% CI: 43.9% - 48.9%) in rural areas, and 18.5% (95% CI:16.2%-21.1%) in urban areas (Table 1). Figure 1 (right panel) presents the contemporary malaria situation in survey locations of the country and can be used for malaria interventions in Mozambique.

Fig 1. Study clusters of the 2018 MMIS (left), and cluster level RDT prevalence (right panel)

Compared with the neighboring countries, the prevalence of malaria in Mozambique was more than twofold higher (Table 2).

The prevalence of malaria varies from province to province: lowest in Maputo (1%) and higher in Cabo Delgadi (57.3%) provinces of the country (Table 1, Fig 2, left panel).

Fig 2. Observed malaria prevalence at different provinces of Mozambique (left panel) and country altitude (right panel).

The results of geostatistical model which took into account the spatial correlation and non-spatial multilevel analysis by including survey design weights are given in Table 3.

From the geostatistical model, place of residence, mothers educational level, child age, household wealth index, child anaemia level, cluster-level malaria incidence rate and ITN-coverage were found significantly associated with malaria infection. In the geostatistical modeling part an exponential correlation function was assumed. The assumption ~~provide~~ proved to be correct as the correlation function is supported by the empirical variogram (Figure 3).

Fig 3. Plots from variogram diagnostic check for the presence of residual spatial correlation (left-hand panel) and for compatibility of the data with the fitted geostatistical model 4 (right-hand panel). The solid line is the empirical variogram of the data. The shaded areas are 95% tolerance bands under the hypothesis of spatial independence (left-hand panel) and under the fitted model 4, (right-hand panel).

For a child living in a rural area, the odds of being malaria parasitaemia is 2.9 times as large as the odds of a child living in urban areas. The odds of being malaria-positive for anaemic child is 3.5 times that of non-anaemic child. Further, the odds of being malaria-positive for a child whose mother education level is secondary or higher is 0.64 times less likely compared with a child whose mothers are not educated.

The likelihood of being parasitaemia for children living with middle and better household wealth index were less likely compared with children living with poor(est) wealth index. Child sex and the availability of bed nets in the household were not significantly related with malaria prevalence ($P - value > 0.05$).

Compared with the non-spatial variation ($\tau^2 = 0.508$) the spatial variation is higher ($\sigma^2 = 0.697$). In the standard non-spatial multilevel modeling, the variance of the random intercept which corresponds to the cluster-level variability is 0.844. Using the non-spatial model the intraclass correlation is 0.204, ($\frac{0.844}{0.844+3.29} = 0.204$), implying that two subjects located in the same cluster had a correlation equal to 0.204 to be parasitaemia.

Figure 4 presents spatial predictions over the study locations, fixing the model parameters at the MCML estimates without any covariate. It also provides the predictive distribution of prevalence in each grid cell through the marginal prevalence (left panel) and probability that the estimated prevalence is above 20% (right panel).

Table 1. RDT prevalence of children of age 6-59 months classified as having malaria by different background characteristics and relative change between 2011 DHS and 2018 MMIS

3.

Factors	2011 DHS			2018 MIS		
	n	**Proportion**	**95% CI**	n	**Proportion**	**95% CI**
**Residence**						
Urban	1599	0.167	(0.146,0.191)	1540	0.185	(0.162, 0.211)
Rural	3317	0.537	(0.442,0.483)	2807	0.464	(0.439, 0.489)
**Province**						
Niassa	434	0.521	(0.426,0.531)	446	0.483	(0.427, 0.539)
Cabo Delgado	413	0.472	(0.476,0.579)	398	0.573	(0.519, 0.625)
Nampula	411	0.433	(0.512,0.620)	434	0.478	(0.428, 0.529)
Zambezia	594	0.548	(0.409, 0.495)	457	0.445	(0.387, 0.505)
Tete	455	0.364	(0.583,0.684)	373	0.295	(0.245, 0.351)
Manica	480	0.279	(0.676,0.760)	510	0.473	(0.424, 0.522)
Sofala	645	0.305	(0.655,0.732)	487	0.293	(0.254, 0.336)
Inhambane	342	0.365	(0.578,0.689)	352	0.351	(0.300, 0.405)
Gaza	404	0.215	(0.740,0.823)	374	0.169	(0.129, 0.217)
Maputo Province	388	0.032	(0.944,0.982)	297	0.012	(0.004, 0.035)
Maputo City	350	0.0145	(0.965,0.994)	219	0.009	(0.002, 0.036)
**Mother education ~~label~~**						
No education	1538	0.467	(0.437,0.496)	908.00	0.52	(0.483, 0.562)
Primary	2261	0.383	(0.359,0.408)	1960	0.423	(0.395, 0.452)
Secondary/higher	1117	0.228	(0.199,0.261)	858	0.152	(0.114, 0.199)
**Child age(years**						
1	565	0.25	(0.208,0.297)	514	0.33	(0.277, 0.388)
2	1156	0.396	(0.362,0.431)	923	0.367	(0.326, 0.411)
3	1076	0.368	(0.334,0.403)	1000	0.429	(0.391, 0.470)
4	1089	0.416	(0.381,0.451)	933	0.379	(0.338, 0.422)
5	1030	0.414	(0.378,0.451)	977	0.414	(0.373, 0.456)
**Child sex**						
Male	2406	0.394	(0.371,0.417)	2154	0.397	(0.368, 0.425)
Female	2510	0.368	(0.346,0.392)	2193	0.381	(0.354, 0.408)
**HH Mosquito bednet**						
No	1751	0.403	(0.375,0.431)	388	0.483	(0.421, 0.620)
Yes	3165	0.369	(0.348,0.389)	3959	0.379	(0.359, 0.399)
**Wealth index**						
Poorest	874	0.548	(0.510,0.586)	852	0.58	(0.539, 0.620)
Poor	937	0.515	(0.478,0.553)	865	0.517	(0.476, 0.559)
Middle	955	0.412	(0.376,0.450)	815	0.422	(0.375, 0.469)
Rich	1100	0.257	(0.227,0.290)	988	0.207	(0.177, 0.241)
Richest	1050	0.054	(0.040,0.074)	827	0.028	(0.017, 0.047)
**Anaemia level**						
Non-anaemic	1699	0.478	(0.148,0.193)	974	0.218	(0.181, 0.259)
Anaemic	3217	0.169	(0.457,0.498)	3369	0.434	(0.412, 0.456)
**Total/National Level**	**4916**	**0.381**	**(0.365,0.397)**	4347	0.389	(0.369,0.408)

Table 2. Prevalence of malaria in children under five years from recent surveys in neighboring countries of Mozambique

Neighboring country	Survey year & data source	Child malaria prevalence(%)
Tanzania	MIS 2017	7.312
Malawi	MIS 2017	15.2.203
Zambia	MIS 2018	9.011
Zimbabwe	MIS 2016	0.202

Areas with greater than 80% probability of exceeding the threshold were considered hotspots. Central and Northern provinces of Mozambique have locations with predicted prevalence above 20%. The dark green areas show locations where prevalence is above 20%, at 80% certainty.

Fig 4. Malaria prevalence predictions among children aged under five year in Mozambique (left panel) and exceedance probabilities (right panel) for the MMIS data.

4 Discussion

This study undertook geostatistical analysis of the 2018 MMIS data to identify determinant factors of malaria risk. It also produced contemporary malaria risk maps of Mozambique for children under five years of age across survey clusters and regions of Mozambique. The generated spatially referenced malaria risk map is the first of its kind for Mozambique from a nationally representative geographically-referenced malaria indicator survey data. The map produced illustrates an important synopsis of prevalence of malaria in the country. Therefore, the observed predicted maps can serve as a resourceful tool in planning interventions and a reference point in evaluating their impact across different administrative regions of the country. The predicted map (Figure 4) show that, the health impact of malaria is higher in the northern and lowest in the southern part of the country. This Figure shows that most areas in the northern part of the country are well above a threshold of 20% prevalence. This implies that in these areas, control efforts towards malaria elimination can be considered. For malaria eradication programme implementers, control efforts in these areas would be on reducing transmission through preventive interventions such as mass bed-net distribution and/or indoor residual spraying campaigns. Thus, in the identified high transmission areas, control efforts would need to be more targeted and tailor-made as opposed to universal coverage effort, in order to cut transmission as much as possible. On the other hand, a number of localities in southern part of the country have prevalence below 20% which require less resource to eradicate malaria s compared with the northern part of the country. The following regions: Tete, Gaza, Maputo and Maputo city have locations where predicted malaria prevalence in children under 5 years is less than 20%.

Malaria risk maps generated by ([4,25]) relied on historical survey data and do not reflect current malaria prevalence situation for under-five year children in Mozambique. Further, the analyses done by ([5,7,22,23]) focus on mapping the prevalence of malaria in a certain specific province which does not represent national burden of malaria risk in children under-five years of age. The study conducted by ([48]) does not represent the contemporary situation of malaria in Mozambique.

As presented in other similar studies conducted to analyze malaria indicator survey datasets ([14,15,18,19]) the likelihood of having malaria increased as a child gets older. This positive relationship between malaria and child age showed that, the older the

Table 3. Parameter estimates from the geostatistical model (4) of malaria prevalence in children under five years of age in Mozambique

**Factors**	AOR	95% AOR CI
$\hat{\beta}_0$	0.366	(0.195,0.684)
Child level factors		
Age (in moth)	1.016	(1.010,1.021)
**Sleep under bed net** (ref: No)	0.736	(0.557,0.971)
**Anemia** (ref:not anemic)	3.502	(2.809,4.367)
Household level factors		
**Wealth index**(ref:Poorest)		
Poor	0.999	(0.801,1.246)
Middle	0.674	(0.534,0.852)
Rich	0.523	(0.396,0.691)
Richest	0.186	(0.111,0.313)
**Mother education** (ref:No education)		
Primary	0.868	(0.712,1.053)
Secondary/higher	0.635	(0.455,0.887)
Cluster level factors		
**Residence** (ref:urban)		
Rural	2.902	(2.316,3.636)
**Province**(ref:Cabo Delgado)		
Niassa	0.743	(0.506,1.090)
Nampula	1.011	(0.714,1.429)
Zamboza	0.808	(0.579,1.130)
Tete	0.373	(0.243,0.572)
Manica	0.833	(0.587,1.182)
Sofala	0.605	(0.421,0.870)
Inhambane	1.277	(0.862,1.891)
Gaza	0.138	(0.084,0.227)
Maputo Province	0.021	(0.007,0.060)
Maputo City	0.032	(0.007,0.143)
**ITN coverage**	0.25	(0.110,0.567)
**Malaria incidence**	4.005	(1.765,9.088)
**Spatial Covariance Prams**		
σ^2	0.697	0.396
ϕ	0.803	0.564
τ^2	0.508	1.164

AOR stands for adjusted odds ratio, and CI for confidence interval.

child the higher chance to be infected by malaria. This may be due to declining breast feeding which exposes a child to maternal antibodies.

~~The 95% CI for estimated adjusted odds-ratio obtained using the geostatistical model 4 was narrow compared to results obtained from weighted multilevel model 3 (see Table 3). Further, educational level of the mother, sleeping under bednet, and place of residence were found significant in the geostatistical model but not in the non-spatial multilevel analysis.~~

We observed that prevalence estimates vary across different socio-demographic groups as well as different regions of the country. The highest prevalence was observed in Cabo Delgado (57.3% with 95% CI: 51.9-62.5%), Niassa (48.3% with 95% CI: 42.7-53.9%), and Nampula (47.8% with 95% CI: 42.8-52.9%) provinces of the country. This may be due to the fact that, the most populous provinces Zambezia and Nampula have the worst education and health outcomes; and in general northern provinces have worse infrastructure; higher levels of environmental degradation and less economic activity than the south [49]. Further, a secondary school access is unevenly distributed among the provinces and lowers in Cabo Delgado and Niassa province [50].

The lowest prevalence was observed in Maputo province and Maputo city (Table 1). Children living in urban areas had significantly lower risk of having malaria compared with children in rural areas. Children who live in rural areas were 2.9 times more likely to have malaria than those who live in urban areas (adjusted odds ratio (AOR) = 2.902). Similar results were also found in other studies ([13, 18, 51]). Among many other factors, malaria plays a major causative role of anaemia globally ([11, 52–55]). In previous studies, the overall prevalence of anaemia in under-five children in Mozambique was above 70% ([48]). In this study, we found that anaemic children were found 3.5 times higher to be parasitaemia than non-anemic children.

The positive relationship with age indicated that the older the child the higher the risk of contacting malaria. This findings agrees with results reported from analyses of MIS data in Nigeria ([11]), Angola ([12]), Tanzania ([13]), Burkina Faso ([15]), The Gambia ([40]),Cote d'Ivoire ([51]), Malawi ([20]) and Uganda ([18]).

Recently, Amoah and his colleagues ([56]) studied the impact of malaria on child growth using 20 Demographic and Health Surveys conducted in 13 African countries. Their result reveals' that malaria had a significant negative effect on child growth.

In line with other similar studies ([11–13, 17, 18, 40]), our study findings showed that malaria prevalence is strongly associated with mothers' education level, child age, wealth index, cluster level malaria incidence rate, and place of residence. Household wealth index and mothers' educational level were negatively associated with the prevalence of malaria, suggesting that the higher the wealth index and the higher mothers' educational level the lower the risk of acquiring malaria. Supporting our findings, [12, 13] and [18] found a decreased risk for malaria among children living with better household wealth index, and higher mothers education.

The results presented in this study should be considered in light of some limitations. Since the analysis result in this study derived from a nationwide cross-sectional malaria indicator survey, sub-national variations in risk and epidemiological transitions could be triangulated with additional routine data from health information systems and malaria hospitalization. Further, employing model-based geostatistical methods to interpolate information on malaria prevalence at province/locality levels is less perfect when compared to complete, reliable routine data on the monthly presentation of parasitologically diagnosed fevers to health facilities. Since malaria is environmentally mediated infectious disease, in future studies, considering environmental factors [21, 57] in the covariance structure of the model will yield better prediction map.

5 Conclusion

Among other health problems, malaria remains one of the biggest public health problems in Mozambique. Thus, evidence-based interventions are needed to reduce the economic burden ([58–60]), and malaria related diseases in the country. In this respect, the results of this study are useful to make geographically targeted interventions.

The results of study showed that high spatial variation in malaria risk were observed across provinces with higher prevalence in the northern part and lower in the southern part of the country. Children living in urban areas had the lowest risk of infection compared with children living in rural areas indicating that more efforts is needed in those areas. Furthermore our result revealed that malaria risk is linked with child age, household wealth index, mother's educational level, place of residence and child's anaemia level. Moreover, household level determinants of malaria infection that are identified by malaria prevalence maps at cluster and province level could be used in malaria control implementing programs to identify priority intervention areas.

Acknowledgment

The author thanks Professor Eshetu Wencheko, Dr. Birhanu Teshome, and anonymous reviewers for their valuable comments. The paper has been considerably strengthened by their comments.

References

1. WHO. Tailoring malaria interventions in the COVID-19 response; 2020.
2. WHO. Malaria eradication: benefits, future scenarios & feasibility: A report of the Strategic Advisory Group on Malaria Eradication.; 2020.
3. WHO. World malaria report 2018; 2018.
4. Bhatt S, Weiss D, Cameron E, Bisanzio D, Mappin B, Dalrymple U, et al. The effect of malaria control on Plasmodium falciparum in Africa between 2000 and 2015. Nature. 2015;526(7572):207–211.
5. Zacarias O, Andersson M. Spatial and temporal patterns of malaria incidence in Mozambique. Malaria Journal. 2011;10(189).
6. PMI. President's Malaria Initiative (PMI): Malaria Operational Plan FY 2019; 2019.
7. Ferrao J, Niquisse S, Mendes PM JM. Mapping and Modelling Malaria Risk Areas Using Climate, Socio-Demographic and Clinical Variables in Chimoio, Mozambique. International Journal of Environmental Research and Public Health. 2018;15(795).
8. Nyarko S, Cobblah A. Socio-demographic determinants of malaria among under-five children in Ghana. Malar Res Treat. 2014;.
9. Afoakwah C, Deng X, Onur I. Malaria infection among children under five: the use of large-scale interventions in Ghana. BMC Public Health. 2018;18(536).
10. Roberts D, Matthews G. Risk factors of malaria in children under the age of five years old in Uganda. Malaria Journal. 2016;15(246).

11. Adigun AB, Gajere EN, Oresanya O, Vounatsou P. Malaria risk in Nigeria: Bayesian geostatistical modelling of 2010 malaria indicator survey data. Malaria Journal. 2015;14(156).

12. Gosoniu L, Veta AM, Vounatsou P. Bayesian Geostatistical Modeling of Malaria Indicator Survey Data in Angola. PLOS ONE. 2010;5(3).

13. Gosoniu L, Msengwa A, Lengeler C, Vounatsou P. Spatially explicit burden estimates of malaria in Tanzania: bayesian geostatistical modeling of the malaria indicator survey data. PloS One. 2012;7(5).

14. Riedel N, Vounatsou P, Miller JM, Gosoniu L, Chizema-Kawesha E, Mukonka V, et al. Geographical patterns and predictors of malaria risk in Zambia: Bayesian geostatistical modelling of the 2006 Zambia national malaria indicator survey (ZMIS). Malaria Journal. 2010;9(37).

15. Samadoulougou S, Maheu-Giroux M, Kirakoya-Samadoulougou F, De Keukeleire M, Castro M, Robert A. Multilevel and geo-statistical modeling of malaria risk in children of Burkina Faso. Parasites & Vectors. 2014;7(1):350.

16. Khagayi S, Amek N, Bigogo G, Odhiambo F, Vounatsou P. Bayesian spatio-temporal modeling of mortality in relation to malaria incidence in Western Kenya. PLoS ONE. 2014;12(17):e0180516.

17. Kazembe LN, Kleinschmidt I, Holtz TH, Sharp BL. Spatial analysis and mapping of malaria risk in Malawi using point-referenced prevalence of infection data. International Journal of Health Geographics. 2006;5(41).

18. Ssempiira J, Nambuusi B, Kissa J, Agaba B, Makumbi F, Kasasa S, et al. Geostatistical modelling of malaria indicator survey data to assess the effects of interventions on the geographical distribution of malaria prevalence in children less than 5 years in Uganda. PLoS ONE. 2017;12(4:):e0174948.

19. Yankson F, Anto E, Chipeta M. Geostatistical analysis and mapping of malaria risk in children under 5 using point-referenced prevalence data in Ghana. Malaria Journal. 2019;18(67).

20. Chipeta M, Giorgi E, Mategula D, et al. Geostatistical analysis of Malawi's changing malaria transmission from 2010 to 2017. Wellcome Open Research. 2019;4(57).

21. Ejigu B, Wencheko E, Moraga P, Giorgi E. Geostatistical methods for modelling non-stationary patterns in disease risk. Spatial Statistics. 2020;35(100397).

22. Zacarias O, Majlender P. Comparison of infant malaria incidence in districts of Maputo province, Mozambique. Malaria Journal. 2011;10(93).

23. Giardina F, Jonas Franke J, Vounatsou P. Geostatistical modelling of the malaria risk in Mozambique: effect of the spatial resolution when using remotely-sensed imagery. Geospatial Health. 2015;10(333).

24. Hay SI, Snow RW. The Malaria Atlas Project: Developing Global Maps of Malaria Risk. PLoS Med. 2006;3(12):e473.

25. Gething PW, Patil AP, Smith DL, Guerra CA, Elyazar IR, Johnston GL, et al. A new world malaria map: Plasmodium falciparum endemicity in 2010. Malaria Journal. 2011;10(378).

26. Instituto, Nacional, de Saúde. Inquérito Nacional sobre Indicadores de Malária em Moçambique 2018; 2019.

27. Benjamin M, Fish TD, Eitelberg D, Dontamsetti T. The DHS Program Geospatial Covariate Datasets Manual (Second Edition); 2018.

28. da Saude M. Moçambique Inquérito Demográfico e de Saúde 2011; 2011. Available from: `http://dhsprogram.com/pubs/pdf/FR266/FR266.pdf`.

29. Carle AC. Fitting multilevel models in complex survey data with design weights: recommendations. BMC Medical Research Methodology. 2009;9:49–62.

30. Agresti A, Coull BA. Approximate is better than 'exact' for interval estimation of binomial proportions. The American Statistician. 1998;52:119–126.

31. Wilson E. Probable inference, the law of succession, and statistical inference. Journal of the American Statistical Association. 1927;22:209–212.

32. Kott PS, Carr DA. Developing an Estimation Strategy for a Pesticide Data Program. Journal of Official Statistics. 1997;13(4):367–383.

33. Franco C, Little RA, Louis TA, Slud EV. Comparative Study of Confidence Intervals for Proportions in Complex Sample surveys. Journal of Survey Statistics and Methodology. 2019;7:334–364.

34. Rabe-Hesketh S, Skrondal A. Multilevel modelling of complex survey data. Royal Statistical Society (Series A). 2006;169(4):805–827.

35. Asparouhov T. General Multi-Level Modeling with Sampling Weights. Communications in statistics -Theory and methods. 2006;35(3):93–103.

36. Grilli L, Pratesi M. Weighted estimation in multilevel ordinal and binary models in the presence of informative sampling designs. Survey Methodology. 2004;30:93–103.

37. Pfeffermann D. Modelling of complex survey data: why mode? Why is it a problem? How can we approach it? Survey Methodology. 2011;37:115–136.

38. Pfeffermann D, Skinner CJ, Holmes DJ, Goldstein H, Rasbash J. Weighting for unequal selection probabilities in multilevel models. Royal Stat Soc. 1998;60:23–40.

39. Diggle P, Tawn J, Moyeed R. Model-based geostatistics. Journal of the Royal Statistical Society Series C (Applied Statistics). 1998;47:299–350.

40. Diggle P, R M, Rowlingson B, Thomson M. Childhood Malaria in the Gambia: A Case-Study in Model-Based Geostatistics. Journal of the Royal Statistical Society Series C (Applied Statistics). 2002;51(4):493–506.

41. Stein M. Interpolation of Spatial Data: Some Theory for Kriging. New York: Springer; 1999.

42. Matern B. Spatial Variation. Berlin: Springer-Verlag; 1986.

43. Geyer CJ, Thompson EA. Constrained Monte Carlo Maximum Likelihood for Dependent Data. Journal of the Royal Statistical Society B. 1992;54(3):657–699.

44. Geyer CJ. On the Convergence of Monte Carlo Maximum Likelihood Calculations. Journal of the Royal Statistical Society B. 1994;56(1):261–274.

45. Giorgi E, Diggle PJ. PrevMap: an R package for prevalence mapping. Journal of Statistical Software. 2017;78(8):1–29.

46. R Core Team. R: A Language and Environment for Statistical Computing; 2018. Available from: https://www.R-project.org/.

47. StataCorp. Stata Statistical Software: Release 14; 2015. Available from: https://www.stata.com/.

48. Mabunda S, Casimiro S, Quinto L, Alonso P. A country-wide malaria survey in Mozambique. I. Plasmodium falciparum infection in children in different epidemiological settings. Malaria Journal. 2008;7(216).

49. SIDA. MOZAMBIQUE MULTIDIMENSIONAL POVERTY ANALYSISSTATUS AND TRENDS; 2019.

50. Fox L, Santibanez L, Nguyen N, Andre P. Education Reform in Mozambique: Lessons and Challenges; 2012.

51. Raso G, Schur N, Utzinger J, Koudou BG, Tchicaya ES, Rohner Fea. Mapping malaria risk among children in Cote d'Ivoire using Bayesian geo-statistical models. Malaria Journal. 2012;11(160).

52. Korenromp EL, Armstrong-Schellenberg JR, Williams BG, Nahlen BL, Snow RW. Impact of malaria control on childhood anaemia in Africa: a quantitative review. Trop Med Int Health. 2004;9(10):1050–1065.

53. Naing C, Sandhu NK, Wai VN. The Effect of Malaria and HIV Co-Infection on Anemia: A Meta-Analysis. Medicine. 2016;95(14).

54. Kassebaum NJ, Jasrasaria R, M N, Wulf SK, Johns N. A systematic analysis of global anemia burden from 1990 to 2010. Blood. 2014;123(5):615–624.

55. Ejigu B, Wencheko E, Berhane K. Spatial pattern and determinants of anaemia in Ethiopia. PLoS ONE. 2018;13(5):e0197171.

56. Amoah B, Giorgi E, Heyes DJ, Burren S, Diggle P. Geostatistical modelling of the association between malaria and child growth in Africa. International Journal of Health Geographics. 2017;17(7).

57. Ejigu B, Wencheko E. Introducing Covariate Dependent Weighting Matrices in Fitting Autoregressive Models and Measuring Environmental Autocorrelation. Spatial Statistics. 2020;38(100454).

58. Aryeetey GC, Agyemang SA, Aubyn VN, Aikins M, Bart-Plange CN, Malm KL, et al. Economic burden of malaria on businesses in Ghana: a case for private sector investment in malaria control. Malaria Journal. 2016;15(454).

59. Gallup JL, Sachs JD. The economic burden of malaria. Am J Trop Med Hyg. 2001;64(Suppl 1):85–96.

60. Sicuri E, Vieta A, Lindner L, Constenla D, Sauboin C. The economic costs of malaria in children in three sub -Saharan countries: Ghana, Tanzania and Kenya. Malaria Journal. 2013;12(307).
