# Supplementary material for: Geostatistical analysis and mapping of malaria risk in children of Mozambique
Source: PLoS One. 2020 Nov 9;15(11):e0241680. doi: 10.1371/journal.pone.0241680 (PMC7652261; doi:10.1371/journal.pone.0241680)
Supplement: Attachment — Submitted filename: Response to Reviewers.pdf [file pone.0241680.s003.pdf]

Review Comments to the Author

Reviewer #1: Since the non-spatial model results have been removed from the manuscript in response to my earlier comment, I suggest that the authors consider including them as part of supplementary material. In this way, interested readers can go over to the supplementary material to get more details.

- Model description and results obtained from the non-spatial model have been submitted as supplementary material.

Reviewer #2: Even though the writer of this manuscript is sole author, while writing it, he frequently said "our understanding, our knowledge". Is that appropriate being a sole author?

- Thanks a lot for your valuable comment. Addressed.
